# Comprehensive Review on the Use of Oral Cholera Vaccine (OCV) in Ethiopia: 2019 to 2023

**DOI:** 10.1093/cid/ciae194

**Published:** 2024-07-12

**Authors:** Moti Edosa, Yeonji Jeon, Abel Gedefaw, Dejene Hailu, Edlawit Mesfin Getachew, Ondari D Mogeni, Geun Hyeog Jang, David Mukasa, Biruk Yeshitela, Tomas Getahun, Julia Lynch, Malika Bouhenia, Yeshambel Worku Demlie, Mukemil Hussen, Mesfin Wossen, Mekonnen Teferi, Se Eun Park

**Affiliations:** Public Health Emergency Management, Ethiopia Public Health Institute, Addis Ababa, Ethiopia; Clinical, Assessment, Regulatory, Evaluation (CARE) Unit, International Vaccine Institute, Seoul, Republic of Korea; Clinical, Assessment, Regulatory, Evaluation (CARE) Unit, International Vaccine Institute, Seoul, Republic of Korea; College of Medicine and Health Sciences, Hawassa University, Hawassa, Ethiopia; Clinical, Assessment, Regulatory, Evaluation (CARE) Unit, International Vaccine Institute, Seoul, Republic of Korea; School of Public Health, Hawassa University, Hawassa, Ethiopia; Clinical Trials Directorate, Armauer Hansen Research Institute, Addis Ababa, Ethiopia; Clinical, Assessment, Regulatory, Evaluation (CARE) Unit, International Vaccine Institute, Seoul, Republic of Korea; Biostatistics and Data Management (BDM) Department, International Vaccine Institute, Seoul, Republic of Korea; Biostatistics and Data Management (BDM) Department, International Vaccine Institute, Seoul, Republic of Korea; Bacterial and Viral Disease Research Directorate, Armauer Hansen Research Institute, Addis Ababa, Ethiopia; Clinical Trials Directorate, Armauer Hansen Research Institute, Addis Ababa, Ethiopia; Cholera Program Director, International Vaccine Institute, Seoul, Republic of Korea; Global Task Force on Cholera Control (GTFCC), World Health Organization (WHO), Geneva, Switzerland; Public Health Emergency Management, Ethiopia Public Health Institute, Addis Ababa, Ethiopia; Public Health Emergency Management, Ethiopia Public Health Institute, Addis Ababa, Ethiopia; Public Health Emergency Management, Ethiopia Public Health Institute, Addis Ababa, Ethiopia; Clinical Trials Directorate, Armauer Hansen Research Institute, Addis Ababa, Ethiopia; Clinical, Assessment, Regulatory, Evaluation (CARE) Unit, International Vaccine Institute, Seoul, Republic of Korea; Department of Global Health and Disease Control, Yonsei University Graduate School of Public Health, Seoul, Republic of Korea

**Keywords:** Ethiopia, Cholera, OCV, global OCV stockpile, OCV dosing schedules

## Abstract

**Background:**

Cholera outbreaks in Ethiopia necessitate frequent mass oral cholera vaccine (OCV) campaigns. Despite this, there is a notable absence of a comprehensive summary of these campaigns. Understanding national OCV vaccination history is essential to design appropriate and effective cholera control strategies. Here, we aimed to retrospectively review all OCV vaccination campaigns conducted across Ethiopia between 2019 and 2023.

**Methods:**

The OCV request records from 2019 to October 2023 and vaccination campaign reports for the period from 2019 to December 2023 were retrospectively accessed from the Ethiopia Public Health Institute (EPHI) database. Descriptive analysis was conducted using the retrospective data collected.

**Results:**

From 2019 to October 2023, Ethiopian government requested 32 044 576 OCV doses (31 899 576 doses to global stockpile; 145 000 doses to outside of stockpile). Around 66.3% of requested doses were approved; of which 90.4% were received. Fifteen OCV campaigns (12 reactive and 3 pre-emptive) were conducted, including five two-dose campaigns with varying dose intervals and single-dose campaigns partially in 2019 and entirely in 2021, 2022 and 2023. Overall vaccine administrative coverage was high; except for Tigray region (41.8% in the 1st round; 2nd round didn't occur). The vaccine administrative coverage records were documented, but no OCV coverage survey data was available.

**Conclusions:**

This study represents the first comprehensive review of OCV campaigns in Ethiopia spanning the last five years. Its findings offer valuable insights into informing future cholera control strategies, underscoring the importance of monitoring and evaluation despite resource constraints. Addressing the limitations in coverage survey data availability is crucial for enhancing the efficacy of future campaigns.

## INTRODUCTION

Vaccination is a feasible rapid intervention to prevent the emergence of cholera and contain further spread during outbreaks [1]. Among three World Health Organization (WHO)-prequalified oral cholera vaccines (OCVs) (Dukoral®, Shanchol^™^, and Euvichol-Plus®), Shanchol^™^ and Euvichol-Plus® have been supplied to the global OCV stockpile and used for mass vaccination campaigns [2]. A Gavi supported global stockpile of OCV was established in 2013 to support the OCV use in outbreak responses (through the International Coordinating Group [ICG] approval process) [1]. In 2017, the “Ending Cholera: A Global Roadmap to 2030” was launched and the use of OCV was expanded to include preventive campaigns with approval of requests initially made by the WHO Global Task Force on Cholera Control (GTFCC) [3]. Currently, access to OCV for preventive campaigns requires a multi-year plan and application through Gavi [4]. Since the 2019 cholera outbreaks, the Ethiopian government has submitted several official requests to the ICG for reactive and preventive use of OCV doses from the global stockpile [5] and an additional request was made to the government of the Republic of Korea bilaterally in response to the mass cholera outbreaks [6]. In parallel, the “Multi-sectoral Cholera Elimination Plan, Ethiopia 2022–2028' was developed by the Ethiopian government, which has been endorsed nationally and announced at the 75th World Health Assembly in 2022; encompassing the potential future use of OCVs based on the mapping of cholera “hotspots” [7].

In 2019, GTFCC OCV Working Group published a review paper on the global OCV use for 5 years, since the establishment of stockpile in 2013, to draw lessons from global OCV deployments and related campaigns [8]. The review paper demonstrated that many countries were using OCVs for both outbreak response and endemic cholera prevention with high coverage in general [8]. However, some campaigns showed relatively lower coverage in adult males and some decreased coverage was observed during the second round of vaccination [8]. In addition, there was a delay between onset of outbreak and implementation of vaccination and/or between the first round and second round [8]. According to this report, Ethiopia is one of the countries that received more than 1 million doses of OCV from the global stockpile between 2013 and 2018 [8]. Since 2019, however, the number of received doses has significantly increased to approximately 18 million doses [5]. To trace and understand this increased OCV deployment in Ethiopia, documenting and reviewing OCV campaigns conducted in Ethiopia would be important. The GTFCC cholera research agenda suggests evidence-generation on OCV use, including the community level duration of protection per dosing schedule, the impact of OCV vaccination timing on outbreak prevention and control, and potential delivery strategies to optimize OCV coverage [9]. A comprehensive review of past use of OCVs in the country could provide the groundwork for future research to address these gaps and policies. Moreover, analyzing such vaccination history data including coverage will also provide an opportunity to identify barriers to uptake and characterize acceptance towards cholera vaccines [10, 11], and lessons learned regarding feasibility and efficiency of strategies, and ultimately these impacts could improve future campaigns.

Here we aimed to conduct a comprehensive retrospective review of all OCV vaccination campaigns conducted in Ethiopia from 2019 to 2023. The GTFCC monitoring and reporting guidelines on the use of OCV recommends the tracking of: OCV administrative coverage per vaccination campaigns; proportion of hotspots targeted by the vaccination plans; and the proportion of doses used in campaigns to respond to an outbreak compared to doses administered during preventive campaigns [12]. Our review focused on the OCV doses requested to the ICG by vaccination type, approved, and delivered; OCV dose numbers and dose intervals applied in each campaign and rolled-out per region, zone, and woreda; OCV vaccination periods per campaign; OCV coverage rates per region, zone, and woreda in Ethiopia; and sex and age-stratified information on the OCV vaccinated populations.

## METHODS

### Dataset Description

The OCV request records from 2019 to October 2023 and vaccination campaign reports for the period from 2019 to December 2023 were retrospectively accessed from the Ethiopia Public Health Institute (EPHI) database. The data prior to 2019 were scattered and challenging to obtain and therefore not included in this analysis. Ethiopia officially has 4 levels of administrative division: Region, Zone, Woreda, and Kebele in descending size order. Ethiopia uses an excel-based and paper-based data collection method to document all OCV vaccination-related data at kebele (ward)- and woreda (district)-level, and subsequently compiled into a national database.

Regional level data are collected using the Open Data Kit (ODK) system. This data set included: date and number of OCV doses requested, approved, and received; OCV vaccination campaigns conducted per region, zone, woreda with planned and actual number of doses administered; OCV vaccination administrative coverage based on actual doses administered and number of population targeted per vaccination target areas (based on the latest administrative census); and sex- and age-stratified data on populations administered with cholera vaccines. Because not all kebeles in targeted woredas were always selected for vaccination, total population number per woreda and population of vaccination targeted kebeles were separately reported in the paper.

### Data Analysis

The request numbers refer to each OCV request made by the Ethiopian government to the ICG, according to the EPHI records. For non-stockpile requests, a discernible request number was given in our analysis. Total population of woredas represent population in the year when OCV vaccination campaigns were conducted. In most past OCV campaigns, the target population was the population in kebeles planned for vaccine administration, excluding infants under 1 year old. The OCV administrative coverage was calculated based on the actual number of people vaccinated (numerator) out of the OCV target populations (denominator) in each round of vaccination per region, zone, and woreda levels in Ethiopia.

## RESULTS

### OCV Doses Requested, Approved, Received, and Used in Ethiopia

**Figure 1.**
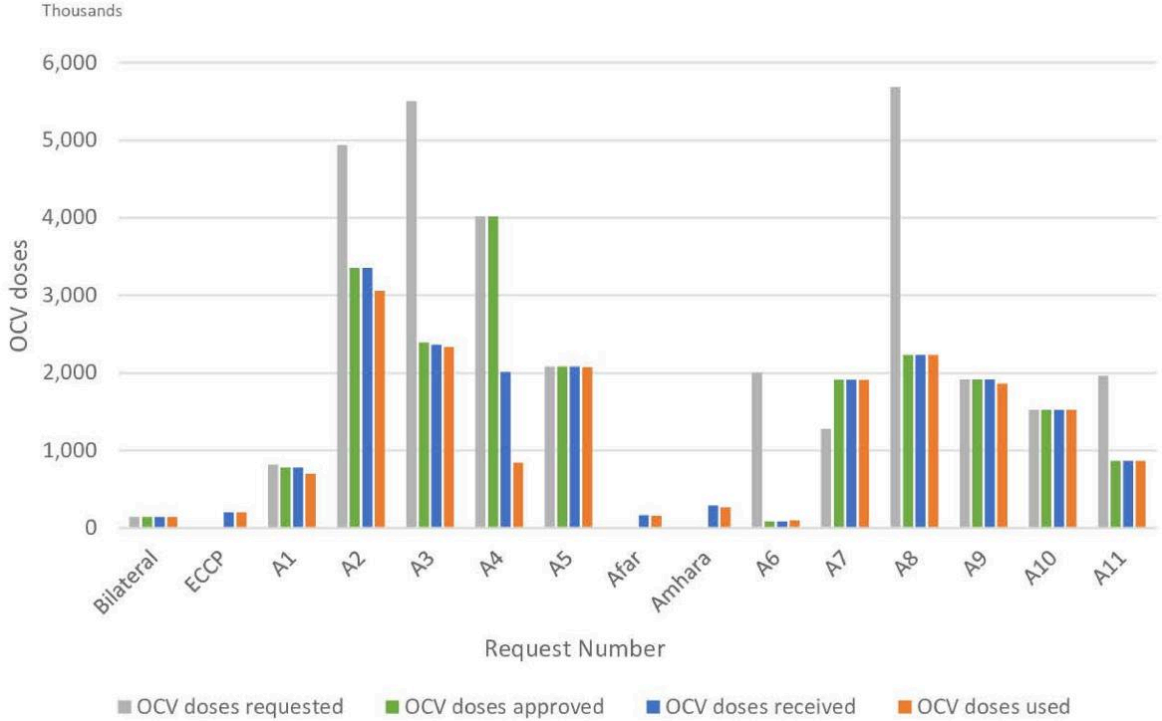
OCV doses made available in Ethiopia from 2019 to October 2023. The figure shows OCV doses requested, approved, and received in Ethiopia from 2019 to October 2023, and doses used in last 5 years. “Request number” refers to each OCV request made by the Ethiopian government to the ICG (A1-A11) and non-stockpile requests (Bilateral, ECCP). “Bilateral” represents the Ethiopian government's bilateral request to the government of the Republic of Korea for OCV doses for cholera outbreak control in 2019. “ECCP” represents “Ethiopia Cholera Control and Prevention” project. “Afar” and “Amhara” represent preventive vaccination campaigns conducted in Afar and Amhara region, respectively using remaining OCV doses from other vaccination campaigns. Abbreviation: OCV, oral cholera vaccine.

From 2019 until October 2023, a total of 32 044 576 OCV doses were requested by the Ethiopian government with 31 899 576 doses (11 requests: A1–A11) to the ICG and 1 request for 145 000 doses through the government of the Republic of Korea. The OCV doses (202 491) used in a preemptive vaccination in May 2022 [13] as part of the earlier planned Ethiopia Cholera Control and Prevention (ECCP) project is not counted in this total number of OCV doses requested to the ICG because these doses were procured as part of a joint research collaboration between the International Vaccine Institute (IVI) and Armauer Hansen Research Institute (AHRI) under the Ministry of Health in Ethiopia. The ICG approved 66.3% (21 148 800/31 899 576) of the requested OCVs and 90.4% (19 113 386/21 148 800) of the approved doses were delivered to Ethiopia; therefore, 59.9% (19 113 386/31 899 576) of the total requested OCV doses were received in Ethiopia (Table 1, Figure 1). The request A6, which was almost immediately after the ICG changed to a single dose recommendation for reactive campaigns in October 2022, shows only 4.3% (86 910/2 000 466) of the requested OCV doses were approved and received in Ethiopia for this outbreak response due to extreme shortage of vaccine in the global stockpile.

**Table 1.** OCV Doses Requested and Made Available in Ethiopia From 2019 to October 2023

OCV Doses Requested and Made Available										
Request Number^a^	Request Type ^b^	OCV Requested		OCV Request Approved		Approved OCV Doses Received in Ethiopia		OCV Doses Used & Leftover		
		OCV Doses Requested (n)	Date of OCV Doses Requested (YYYY/MM/DD)	OCV Doses Approved (n)	Date of OCV Doses Approved (YYYY/MM/DD)	OCV Doses Received at Entry port in Ethiopia^c^ (n)	Date of OCV Doses Received at Entry Port in Ethiopia (YYYY/MM/DD)	OCV Doses Used (n)	OCV Doses Unused Due to VVM Change (n)	OCV Doses Returned Unused and in Good Condition^d^ (n)
**Request outside of Global OCV Stockpile**										
Bilateral^e^	Reactive	145 000	n.a	145 000	n.a	145 000	2019/06/14	145 000	n.a	n.a
ECCP	Preemptive/Preventive	n.a	n.a	n.a	n.a	202 360	2021/11/18	202 491^f^	n.a	n.a
**Total**		**145 000**	**n.a**	**145 000**	**n.a**	**347 360**	**n.a**	**347 491**	**n.a**	**n.a**
**Request to Global OCV Stockpile**										
A1^g^	Preemptive/Preventive	817 705	2019/05/07	778 766	2019/05/10	778 766	2019/05/17 (1st round)^h^ 2019/12/03 (2nd round)	698 970	496	32 311
A2	Reactive	4 938 802	2020/11/16	3 354 278	2020/11/19	3 354 400	2020/12/06 (1st round) 2021/02/21 (2nd round)	3 059 678	22	n.a
A3	Reactive	5 501 446	2021/03/03	2 390 454	2021/03/05	2 363 164	2021/03/18 (1st round) 2021/07/09 (2nd round)	2 332 578	751	26 545
A4	Preemptive/Preventive	4 017 218	2021/05/11	4 017 218	2021/05/13	2 008 650	2021/05/22	840 774	n.a	n.a
A5	Reactive	2 077 959	2021/11/04	2 077 959	2021/11/06	2 077 958	2021/11/18	2 073 728	296	3583
A6	Reactive	2 000 466	2022/11/30	86 910	2022/12/02	86 910	2022/12/15	100 713	n.a	5687
A7	Reactive	1 275 898	2023/03/01	1 910 416	2023/03/29	1 910 416	2023/04/05	1 909 405	53	957
A8	Reactive	5 867 505	2023/06/13	2 230 038	2023/07/09	2 230 038	2023/07/20	2 229 941	68	169
A9	Reactive	1 917 914	2023/08/01	1 917 914	2023/08/03	1 917 914	2023/09/12	1 858 472	85	5389
A10	Reactive	1 522 495	2023/09/29	1 522 495	2023/10/04	1 522 495	2023/10/20	1 522 407	246	1283
A11	Reactive	1 962 168	2023/10/15	862 352	2023/10/17	862 675	2023/10/27	862 326	16	333
**Total**		**31 899 576**	**n.a**	**21 148 800**	**n.a**	**19 113 386**	**n.a**	**17 488 992**	**2033**	**76 257**
**Grand total**		**32 044 576**	**n.a**	**21 293 800**	**n.a**	**19 460 746**	**n.a**	**17 836 483**	**2033**	**76 257**

Abbreviations: ECCP, Ethiopia Cholera Control and Prevention; n.a, not applicable; OCV, oral cholera vaccine; VVM, Vaccine Vial Monitor.

The bold values refer to the total or subtotal amount.

^a^Request number refers to each OCV request made by the Ethiopian government.

^b^Request Type refers to a preemptive/preventive or reactive campaign per the Ethiopian government's OCV request dossiers.

^c^All OCV doses approved were delivered to Addis Ababa, the entry port in Ethiopia.

^d^Remaining OCV doses are returned to Ethiopian Pharmaceuticals Supply Agency (EPSA) for storage and stored doses are used for other vaccination campaigns.

^e^Bilateral: Ethiopian government's bilateral request to the government of the Republic of Korea for OCV doses for cholera outbreak control in 2019.

^g^OCV doses were requested for preemptive/preventive vaccination, but doses were partially used for reactive vaccination and partially for preemptive vaccination at the moment of campaign.

^f^Total 202 491 doses were used: 202 360 doses procured and delivered under ECCP and additional 131 available doses from health centers.

^h^Approved OCV doses were shipped in a separate batch per round according to the campaign schedule in the case of 2 rounds of vaccination.

Generally, all approved doses were delivered to Ethiopia, but the request A4 for reactive vaccination campaign in Tigray region was exceptional (4 017 218 requested and approved; 2 008 650 delivered). When a campaign is designed for 2 rounds, approved OCV doses are usually shipped in a separate batch per round according to the campaign schedule, and OCV doses for second round are delivered after a technical report from first round is submitted to the ICG. In the case of Tigray campaign, the first round's technical report concluded that the second round could not be implemented due to insecurity in the region and the second-round doses were not sent although the doses for 2 rounds had been approved. Of the total 31 899 576 doses (requests A1–A11) requested, 21 148 800 doses were approved, of which 19 113 386 doses were delivered to Ethiopia and 17 488 992 doses were administered. Based on the available records, 2033 doses were not used due to vaccine vial monitor (VVM) change signifying lack of integrity of the cold chain which occurred in the hard-to-reach areas under conflict. 76 257 unused doses were returned to the Ethiopian Pharmaceuticals Supply Agency (EPSA) for cold chain storage, to be used for future reactive vaccination, such as campaigns conducted in Afar and Amhara region in 2022 (Table 2). Among the gap of 1 546 104 doses between delivered and administered, 1 167 876 doses could not be used in Tigray campaign as planned and have not been retrieved to EPSA yet. The remaining gap of 378 228 doses is attributed to remaining doses from prior campaigns but marked as “not applicable” in the database or some damaged vials which were not described in Table 1.

**Table 2.** OCV Vaccination Campaigns in Ethiopia From 2019 to 2023

Request Number^a^	Region	OCV Target Population^b^	Dose Regimen SD/2D	1st Round				Dose Interval (If 2 Doses Administered)	2nd Round			
				Campaign Start Date (YYYY/MM/DD)	Campaign End Date (YYYY/MM/DD)	Number of People Vaccinated with OCV (n)	Administrative Coverage^c^ (%)		Campaign Start Date (YYYY/MM/DD)	Campaign End Date (YYYY/MM/DD)	Number of People Vaccinated with OCV (n)	Administrative Coverage (%)
**A1**	Oromia	298 343	2D	2019/07/01	2019/07/05	290 740	97.5	24 wks	2019/12/14	2019/12/24	286 203	95.9
	Addis Ababa	17 324	SD	2019/07/01	2019/07/05	17 324	100.0	n.a	n.a	n.a	n.a	n.a
	Afar	92 070	SD	2019/07/01	2019/07/05	81 381	88.4	n.a	n.a	n.a	n.a	n.a
	Sidama	23 322	SD	2019/07/01	2019/07/05	23 322	100.0	n.a	n.a	n.a	n.a	n.a
	**A1-total**	**431 059**	**SD/2D (Oromia)**	**2019/07/01**	**2019/07/05**	**412 767**	**95**.**8**	**24 wks**	**2019/12/14**	**2019/12/24**	**286 203**	**95.9**
**A2**	Gambella	27 128	2D	2020/12/21	2020/12/29	24 226	89.3	9 wks	2021/02/28	2021/03/06	24 226	89.3
	Oromia	503 823	2D	2020/12/21	2020/12/29	483 581	96.0	9 wks	2021/02/28	2021/03/06	482 341	95.7
	Sidama	359 176	2D	2020/12/21	2020/12/29	352 189	98.1	9 wks	2021/02/28	2021/03/06	351 845	98.0
	SNNPR	598 201	2D	2020/12/21	2020/12/29	559 605	93.5	9 wks	2021/02/28	2021/03/06	534 199	89.3
	Somali	113 991	2D	2020/12/21	2020/12/29	124 863	109.5	9 wks	2021/02/28	2021/03/06	122 603	107.6
	**A2-total**	**1 602 319**	**2D**	**2020/12/21**	**2020/12/29**	**1 544 464**	**96**.**4**	**9 wks**	**2021/02/28**	**2021/03/06**	**1 515 214**	**94.6**
**A3**	Oromia	151 227	2D	2021/05/20	2021/05/26	148 201	98.0	12 wks	2021/08/18	2021/08/25	148 201	100.0
	SNNPR	1 044 000	2D	2021/05/20	2021/05/26	1 019 738	97.7	12 wks	2021/08/18	2021/08/25	1 016 438	99.7
	**A3-total**	**1 195 227**	**2D**	**2021/05/20**	**2021/05/26**	**1 167 939**	**97**.**7**	**12 wks**	**2021/08/18**	**2021/08/25**	**1 164 639**	**99.7**
**A4**	**Tigray**	**2 010 680**	**SD**	**2021/06/10**	**2021/06/17**	**840 774**	**41**.**8**	**n.a**	**n.a**	**n.a**	**n.a**	**n.a**
**A5**	Oromia	518 379	2D	2021/12/23	2022/01/01	516 834	99.7	13 wks	2022/03/25	2022/03/31	516 834	99.7
	Somali	520 600	2D	2021/12/23	2022/01/01	520 035	99.9	13 wks	2022/03/25	2022/03/31	520 025	99.9
	**A5-total**	**1 038 979**	**2D**	**2021/12/23**	**2022/01/01**	**1 036 869**	**99**.**8**	**13 wks**	**2022/03/25**	**2022/03/31**	**1 036 859**	**99.8**
**N/A^d^**	Afar	163 544	SD	2022/04/23	2022/04/30	160 340	98.0	n.a	n.a	n.a	n.a	n.a
	Amhara	291 594	SD	2022/05/23	2022/05/31	265 188	90.9	n.a	n.a	n.a	n.a	n.a
	**N/A-total**	**455 138**	**SD**	**n.a**	**n.a**	**425 528**	**93**.**5**	**n.a**	**n.a**	**n.a**	**n.a**	**n.a**
**A6**	Oromia	76 400	SD	2023/01/13	2023/01/20	76 226	99.8	n.a	n.a	n.a	n.a	n.a
	Somali	24 487	SD	2023/01/13	2023/01/20	24 487	100	n.a	n.a	n.a	n.a	n.a
	**A6-total**	**100 887**	**SD**	**2023/01/13**	**2023/01/20**	**100 713**	**99**.**8**	**n.a**	**n.a**	**n.a**	**n.a**	**n.a**
**A7**	Oromia	1 467 612	SD	2023/05/15	2023/05/24	1 466 698	99.9	n.a	n.a	n.a	n.a	n.a
	Somali	442 806	SD	2023/05/15	2023/05/24	442 707	100.0	n.a	n.a	n.a	n.a	n.a
	**A7-total**	**1 910 418**	**SD**	**2023/05/15**	**2023/05/24**	**1 909 405**	**99**.**9**	**n.a**	**n.a**	**n.a**	**n.a**	**n.a**
**A8**	Oromia	571 259	SD	2023/08/10	2023/08/20	571 102	100.0	n.a	n.a	n.a	n.a	n.a
	Sidama	343 158	SD	2023/08/10	2023/08/20	343 156	100.0	n.a	n.a	n.a	n.a	n.a
	SNNPR	1 315 761	SD	2023/08/10	2023/08/20	1 315 683	100.0	n.a	n.a	n.a	n.a	n.a
	**A8-total**	**2 230 178**	**SD**	**2023/08/10**	**2023/08/20**	**2 229 941**	**100**.**0**	**n.a**	**n.a**	**n.a**	**n.a**	**n.a**
**A9**	**Amhara**	**1 867 912**	**SD**	**2023/09/16**	**2023/09/23**	**1 858 472**	**99**.**5**	**n.a**	**n.a**	**n.a**	**n.a**	**n.a**
**A10**	Afar	524 923	SD	2023/11/11	2023/11/21	524 173	99.9	n.a	n.a	n.a	n.a	n.a
	Sidama	365 488	SD	2023/11/11	2023/11/21	365 270	99.9	n.a	n.a	n.a	n.a	n.a
	SNNPR	633 652	SD	2023/11/11	2023/11/21	632 964	99.9	n.a	n.a	n.a	n.a	n.a
	**A10-total**	**1 524 063**	**SD**	**2023/11/11**	**2023/11/21**	**1 522 407**	**99**.**9**	**n.a**	**n.a**	**n.a**	**n.a**	**n.a**
**A11**	Amhara	131 134	SD	2023/11/29	2023/12/05	130 803	99.7	n.a	n.a	n.a	n.a	n.a
	Oromia	731 541	SD	2023/11/29	2023/12/05	731 523	100.0	n.a	n.a	n.a	n.a	n.a
	**A11-total**	**862 675**	**SD**	**2023/11/29**	**2023/12/05**	**862 326**	**100**.**0**	**n.a**	**n.a**	**n.a**	**n.a**	**n.a**
**Grand total**		**15 229 535**	**n.a**	**n.a**	**n.a**	**13 911 605**	**91.3**	**n.a**	**n.a**	**n.a**	**4 002 915**	**97.1**

Abbreviations: n.a, not applicable; OCV, oral cholera vaccine; SD, single dose; SNNPR: Southern Nations, Nationalities, and Peoples’ Region; 2D, 2 doses; wks, weeks.

The bold values refer to the total or subtotal amount.

^a^Request number refers to each OCV request made by the Ethiopian government.

^b^In first round. OCV target population is the population of OCV vaccination targeted kebeles in each woreda excluding infants (ie, < 1 y).

^c^Administrative coverage (%) was calculated based on the actual number of people vaccinated in each round (numerator) out of the OCV target population (denominator) in each round.

^d^It used remaining OCV doses from previous OCV vaccination campaigns.

### OCV Vaccination Dose Intervals and Coverage per Region, Zone, and Woreda in Ethiopia

**Figure 2.**
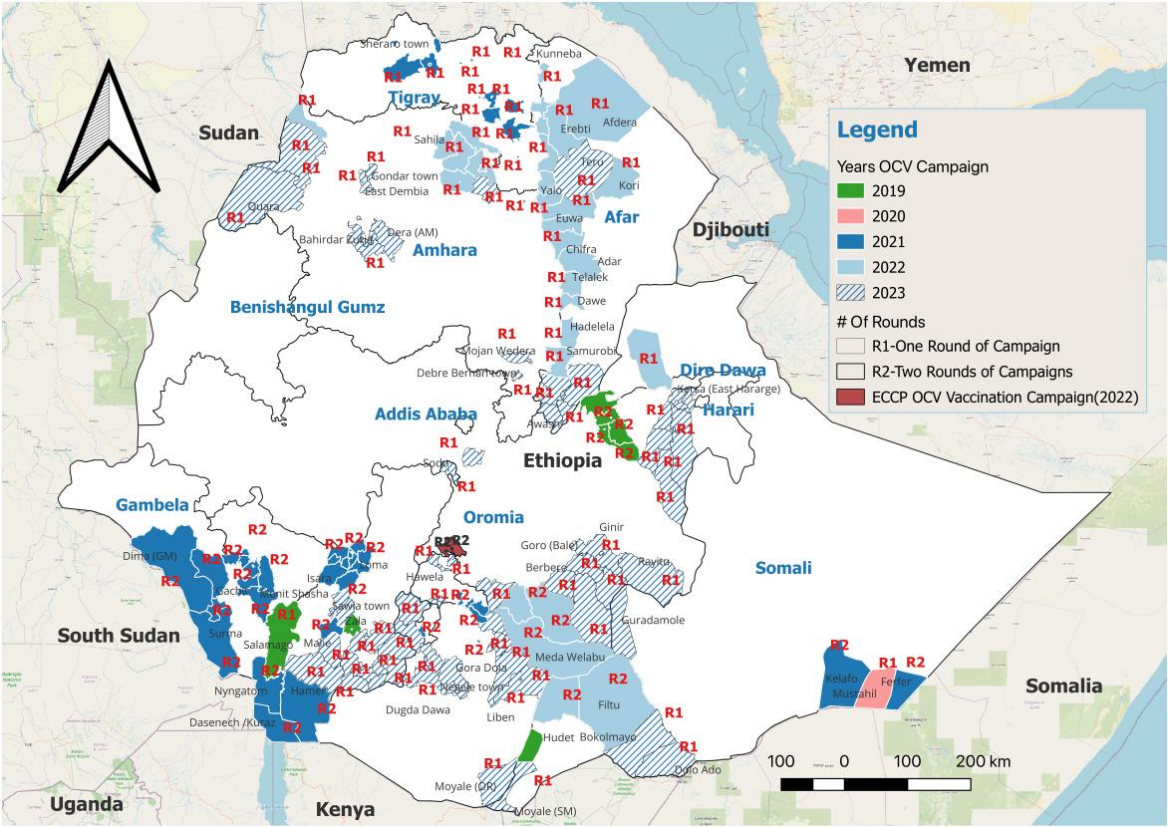
Map of OCV vaccination campaign areas. The map comprehensively shows woredas where OCV vaccination campaigns have been conducted from 2019 to 2023. Number of vaccination rounds conducted in each woreda is represented as either R1 or R2. Abbreviations: ECCP, Ethiopia Cholera Control and Prevention; OCV, oral cholera vaccine.

Based on the OCV doses received from requests A1-A11, 17 488 992 people were vaccinated with either a single dose (SD) or 2-doses (2D) of OCV with different dosing schedules (Table 2, Supplementary Table 1, Figure 2). The OCV doses from request A1 were administered as 2D with 24-week dose interval in Oromia region with 97.5% administrative coverage in first round and 95.9% in second round, but SD strategy was used in other cholera vulnerable regions due to the shortage of the vaccine. OCV doses were requested for pre-emptive vaccination in Tigray region, a site of ongoing conflict. Tigray campaign was conducted in the setting of insecurity or armed conflict, resulting in the lowest OCV administrative coverage across all vaccination campaigns conducted in Ethiopia from 2019 to December 2023 (Table 2). Reactive vaccination campaigns implemented between 2020 and 2021 (request A2, A3, A5) used recommended 2D regimen but dose intervals were more than 2 weeks. In April and May 2022, OCV vaccinations were carried out in Afar and Amhara regions to respond to the outbreak in each region. Leftover doses from other OCV vaccination campaigns conducted prior to April 2022, which have been stored at EPSA, were used for SD strategy to cover more people (Table 2). Six reactive vaccinations in 2023 administered SD reflecting the ICG recommendation in global OCV shortage setting.

### Sex- and Age-Stratified OCV Vaccination in Ethiopia

**Figure 3.**
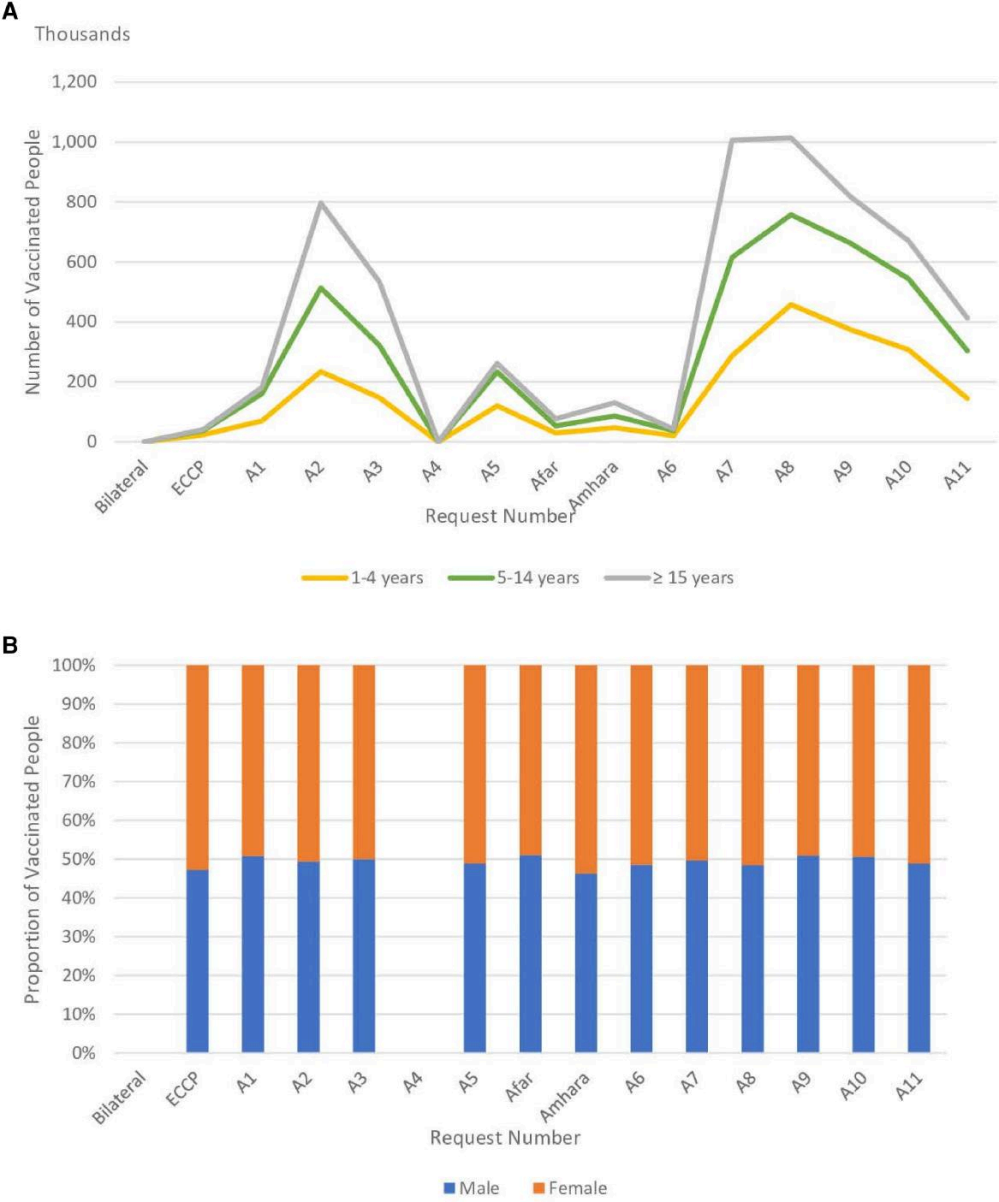

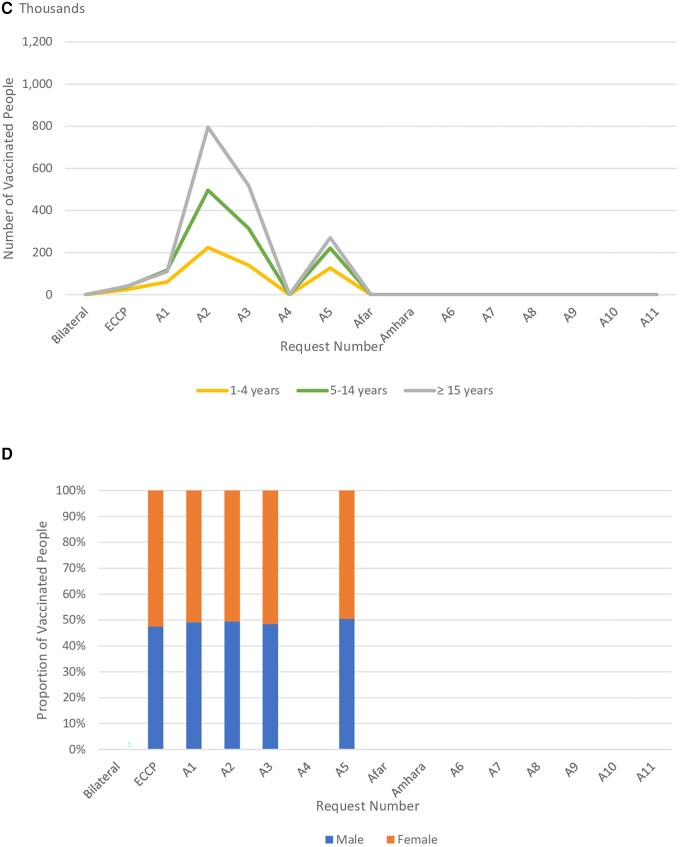
Age group and sex-stratified OCV vaccinated populations from 2019 to 2023. Panels *A*–*D* show age groups and sex of OCV vaccinated populations in each vaccination campaigns. “Request number” refers to each OCV request made by the Ethiopian government to the ICG (A1–A11) and non-stockpile requests (Bilateral, ECCP). “Bilateral” represents the Ethiopian government's bilateral request to the government of the Republic of Korea for OCV doses for cholera outbreak control in 2019. “ECCP” represents “Ethiopia Cholera Control and Prevention” project. “Afar” and “Amhara” represent preventive vaccination campaigns conducted in Afar and Amhara region respectively using remaining OCV doses from other vaccination campaigns. Age and sex stratified data of OCV vaccinated populations from Bilateral and A4 were not available. Afar, Amhara, and A6–A11 administered single dose (SD) strategy; there was no second round information. Panels *A* and *B* exhibit age group and sex stratifications of populations vaccinated respectively in the 1st round; Panels *C* and *D* exhibit age group and sex stratifications of populations vaccinated respectively in the 2nd round. Abbreviation: OCV, oral cholera vaccine.

The sex- and age-stratified data on the OCV vaccinated populations for each vaccination campaign from 2019 to 2023 were described (Table 3, Supplementary Table 2, and Figure 3). There was no sex and age data for the campaign conducted in Southern Nations, Nationalities, and Peoples’ Region (SNNPR) and Somali regions in 2019 through bilateral request and Tigray campaign (request A4). Overall, local populations aged 15 years and older received the largest number of doses of OCV regardless of SD or 2D administration (Table 3), both first (Figure 3*A*) and second round (Figure 3*C*); followed by children aged between 5–14 years and 1–4 years. An exception was in the A1 request related OCV use during the second round of vaccination campaign, where children aged 5–14 years (116 610) were vaccinated slightly more than those aged 15 years and older (109 626) (Table 3 and Supplementary Table 2). The proportion of male and female populations vaccinated with OCV was similar regardless of dosing schedules or campaign types (Figure 3*B* and Figure 3*D*).

**Table 3.** Age-Group and Sex-Stratified Data on OCV Vaccination Campaigns in Ethiopia From 2019 to 2023

Request Number^a^	Region	Number of People Vaccinated With OCV^b^ (n)	1st Round						2nd Round					
			1–4 y^c^		5–14 y^d^		≥15 y		1–4 y		5–14 y		≥15 y	
			M	F	M	F	M	F	M	F	M	F	M	F
**A1**	Oromia	290 740	28 959	28 698	57 610	57 790	58 907	58 776	29 955	30 012	56 963	59 647	53 650	55 976
	Addis Ababa	17 324	405	320	4208	3770	4500	4121	n.a	n.a	n.a	n.a	n.a	n.a
	Afar	81 381	5998	5998	17 861	17 861	20 284	13 379	n.a	n.a	n.a	n.a	n.a	n.a
	Sidama	23 322	0	0	810	800	10 212	11 500	n.a	n.a	n.a	n.a	n.a	n.a
	A1-total	412 767	35 362	35 016	80 489	80 221	93 903	87 776	29 955	30 012	56 963	59 647	53 650	55 976
	**A1-total per age group**		**70 378**		**160 710**		**181 679**		**59 967**		**116 610**		**109 626**	
**A2**	Gambella	24 226	1487	1546	3111	3107	8868	6107	1487	1546	3111	3107	8868	6107
	Oromia	483 581	42 899	43 033	78 596	79 435	116 846	122 772	43 122	43 033	79 954	75 636	117 824	122 772
	Sidama	352 189	18 868	19 933	63 243	63 568	93 039	93 538	19 090	16 931	64 412	65 274	92 017	94 121
	SNNPR	559 605	44 550	43 414	83 773	86 314	147 948	153 606	38 388	39 775	78 105	81 158	145 990	150 783
	Somali	124 863	8789	10 259	23 942	28 166	26 576	27 131	10 190	10 242	20 657	24 635	26 512	30 367
	A2-total	1 544 464	116 593	118 185	252 665	260 590	393 277	403 154	112 277	111 527	246 239	249 810	391 211	404 150
	**A2-total per age group**		**234 778**		**513 255**		**796 431**		**223 804**		**496 049**		**795 361**	
**A3**	Oromia	148 201	8300	8639	24 364	25 358	40 354	41 186	8300	8639	24 364	25 358	40 354	41 186
	SNNPR	1 019 738	57 862	60 192	168 388	175 229	276 990	281 077	56 928	59 251	167 099	173 917	276 769	282 474
	A3-total	1 167 939	66 162	68 831	192 752	200 587	317 344	322 263	65 228	67 890	191 463	199 275	317 123	323 660
	**A3-total per age group**		**134 993**		**393 339**		**639 607**		**133 118**		**390 738**		**640 783**	
**A4**	Tigray	840 774	n.a	n.a	n.a	n.a	n.a	n.a	n.a	n.a	n.a	n.a	n.a	n.a
**A5**	Oromia	516 834	48 959	52 988	93 152	93 264	116 209	112 262	48 975	52 990	93 152	93 255	116 205	112 257
	Somali	520 035	47 940	42 695	98 068	110 178	101 326	119 828	47 944	42 695	98 068	110 186	101 328	119 804
	A5-total	1 036 869	96 899	95 683	191 220	203 442	217 535	232 090	96 919	95 685	191 220	203 441	217 533	232 061
	**A5-total per age group**		**192 582**		**394 662**		**449 625**		**192 604**		**394 661**		**449 594**	
**N/A^e^**	Afar	160 340	15 552	14 026	27 462	25 904	38 888	38 508	n.a	n.a	n.a	n.a	n.a	n.a
	Amhara	265 188	22 908	24 614	40 552	45 939	59 313	71 862	n.a	n.a	n.a	n.a	n.a	n.a
	N/A-total	425 528	38 460	38 640	68 014	71 843	98 201	110 370	n.a	n.a	n.a	n.a	n.a	n.a
	**N/A-total per age group**		**77 100**		**139 857**		**208 571**		**n.a**		**n.a**		**n.a**	
**A6**	Oromia	76 226	7344	7809	13 442	14 526	16 056	17 049	n.a	n.a	n.a	n.a	n.a	n.a
	Somali	24 487	2956	2910	4436	4701	4664	4820	n.a	n.a	n.a	n.a	n.a	n.a
	A6-total	100 713	10 300	10 719	17 878	19 227	20 720	21 869	n.a	n.a	n.a	n.a	n.a	n.a
	**A6-total per age group**		**21 019**		**37 105**		**42 589**		n.a		**n.a**		**n.a**	
**A7**	Oromia	1 466 698	108 619	112 184	233 817	238 020	385 638	388 420	n.a	n.a	n.a	n.a	n.a	n.a
	Somali	442 707	33 352	33 211	71 526	72 158	116 395	116 065	n.a	n.a	n.a	n.a	n.a	n.a
	A7-total	1 909 405	141 971	145 395	305 343	310 178	502 033	504 485	n.a	n.a	n.a	n.a	n.a	n.a
	**A7-total per age group**	…	**287 366**		**615 521**		**1 006 518**		**n.a**		**n.a**		**n.a**	
**A8**	Oromia	571 102	57 544	62 358	97 712	95 258	126 752	131 478	n.a	n.a	n.a	n.a	n.a	n.a
	Sidama	343 156	43 255	44 984	57 601	60 320	64 868	72 128	n.a	n.a	n.a	n.a	n.a	n.a
	SNNPR	1 315 683	122 069	127 788	217 282	229 514	294 082	324 948	n.a	n.a	n.a	n.a	n.a	n.a
	A8-total	2 229 941	222 868	235 130	372 595	385 092	485 702	528 554	n.a	n.a	n.a	n.a	n.a	n.a
	**A8-total per age group**		**457 998**		**757 687**		**1 014 256**		**n.a**		**n.a**		**n.a**	
**A9**	Afar	1 858 472	187 787	187 774	322 197	341 420	437 115	382 179	n.a	n.a	n.a	n.a	n.a	n.a
	**A9-total per age group**		**375 561**		**663 617**		**819 294**		**n.a**		**n.a**		**n.a**	
**A10**	Afar	524 173	52 489	53 289	91 216	96 769	121 130	109 280	n.a	n.a	n.a	n.a	n.a	n.a
	Sidama	365 270	36 547	37 447	61 932	67 787	85 661	75 896	n.a	n.a	n.a	n.a	n.a	n.a
	SNNPR	632 964	63 863	63 663	110 118	116 455	147 337	131 528	n.a	n.a	n.a	n.a	n.a	n.a
	A10-total	1 522 407	152 899	154 399	263 266	281 011	354 128	316 704	n.a	n.a	n.a	n.a	n.a	n.a
	**A10-total per age group**		**307 298**		**544 277**		**670 832**		**n.a**		**n.a**		**n.a**	
**A11**	Amhara	130 803	5156	5563	20 037	20 803	39 435	39 809	n.a	n.a	n.a	n.a	n.a	n.a
	Oromia	731 523	64 779	69 605	131 506	132 057	160 635	172 941	**n.a**	n.a	n.a	n.a	n.a	n.a
	A11-total	862 326	69 935	75 168	151 543	152 860	200 070	212 750	n.a	n.a	n.a	n.a	n.a	n.a
	**A11-total per age group**		**145 103**		**304 403**		**412 820**		**n.a**		**n.a**		**n.a**	
**Grand total per age group**			**2 304 176**		**4 524 433**		**6 242 222**		**609 493**		**1 398 058**		**1 995 364**	

Abbreviations: n.a, not applicable; OCV, oral cholera vaccine; SNNPR: Southern Nations, Nationalities, and Peoples’ Region; y: years.

The bold values refer to the total or subtotal amount.

^a^Request number refers to each OCV request made by the Ethiopian government.

^b^In first round.

^c^12–59 months.

^d^60–179 months.

^e^It used remaining OCV doses from previous OCV vaccination campaigns.

## DISCUSSION

Overall, 15 OCV mass vaccination campaigns were conducted in Ethiopia from 2019 to 2023. 11 campaigns (9 reactive and 2 preemptive campaigns) used OCV doses received through requests made to the global stockpile of OCVs and 2 reactive campaigns in Afar and Amhara region in 2022 used some remaining doses from prior campaigns. OCV doses obtained from outside of global stockpile (a bilateral channel and a research project) were used for 1 reactive campaign and 1 preemptive vaccination, respectively. This preemptive vaccination campaign result under the research project is presented in this CID Supplement [13]. Most of the OCV requests were deployed in response to cholera outbreaks, and dose intervals were more than standard 2 weeks in the case of 2-rounds campaigns. Regardless of dose regimen, all areas targeted showed high administrative coverage except for Tigray region, but formal post-campaign coverage surveys have not been conducted in Ethiopia. Generally, population aged 15 years and older were vaccinated more compared to children aged below 15 years, and there was not much difference between the proportion of sex in vaccinated population. No adverse events following immunization (AEFI) were reported throughout all vaccination campaigns.

During the vaccination campaign implemented in 2019, the Ethiopian government used a SD strategy to cover broader outbreak area due to shortage of the vaccine, except parts of Oromia region where 2D vaccination was rolled-out considering the severity of outbreak in the region [14]. The ICG SD recommendation was announced in October 2022 in response to large scale global cholera epidemics and the shortage in global OCV stockpile [15]. Debates on the effectiveness and impact of SD are ongoing with limited research available thus far. However, some modeling studies demonstrate a speedy vaccination with SD of OCV under a limited vaccine supply may avert more cholera cases and deaths in outbreak setting compared to a standard 2D regimen [16, 17]. Nonetheless, the effectiveness, duration of protection and impact of SD OCV intervention may differ in populations living in different environments with varying degrees of cholera endemicity [18]; as well as between age-groups as children under 5 years may receive limited benefit from an SD [19, 20]. All mass OCV vaccination campaigns conducted in Ethiopia from 2022 used SD strategy (except for the campaign under the ECCP project in May 2022), but no follow-up studies have been performed to investigate SD effectiveness or to explore how long protection would last or optimal timing of catch-up second dose vaccination.

In addition to dose regimen, optimal and feasible dosing schedule is another point to be considered for effective vaccination. The standard dose interval of OCV is 2 weeks [21], but it is not feasible to strictly follow in most emergency settings. Among 5 OCV vaccination campaigns with 2D regimen conducted in Ethiopia from 2019, only 1 vaccination campaign implemented in Shashemene area in May 2022 (under the ECCP research project) adopted the standard 14-day dose interval [13]. The remaining 2D vaccination campaigns showed varying dose intervals from 9 weeks to 24 weeks. Similar to the SD, there are limited studies on OCV dosing schedules. Several studies have explored immunogenicity of alternative dose intervals and found generally comparable immune responses at intervals of 1 or 6 months [22, 23]. A SD strategy may be able to reduce the urgent risk of cholera infection in an ongoing outbreak for the short term; but an extended interval dose is recommended to make protection robust even with delays in the second dose [20, 23, 24]. Nevertheless, this may not be an easy task in the real-world setting. A study conducted in Lusaka, Zambia, suggested the long delay between doses can make people miss an opportunity to get full 2-doses OCV because of population movement [25]. However, further operational research on a longer dosing interval is warranted to answer questions on the effectiveness and impact in real-world settings.

One of the priorities in the GTFCC cholera research agenda is finding vaccination delivery strategies for “hard-to-reach populations” such as internally displaced people (IDP) [9]. This facet is also important to consider because areas of insecurity may be at risk for cholera outbreaks and difficult to provide immunization [26–28]. In Ethiopia, the government conducted campaigns to prevent cholera outbreaks amid the conflict region in Tigray regional state in 2021 [29], targeting populations living in the conflict affected communities and IDP. However, significantly lower vaccination administrative coverage (41.8% in the first round; and second round did not occur) was reported from all woredas in Tigray region during this period as a result of the challenging operational situation on the ground. For instance, barriers to sufficient community engagement and sensitization, limited access to health centers and posts, and management of overall mass vaccination campaigns with proper record keeping and documentations. EPHI uses a mixed vaccination campaign strategy (door-to-door; fixed posts), and campaigns are integrated with other response pillars including surveillance and water, sanitation, and hygiene interventions. Yet it underscores how difficult it can be to implement vaccination effectively in a conflict setting and may infer necessity of identifying and building logistical and operational strategy to equitably administer OCVs to hard-to-reach and vulnerable populations [30].

No post-campaign coverage surveys have been conducted in Ethiopia due to the constraints of manpower and time in the face of the upsurge in outbreaks. Coverage surveys are important to understand the actual coverage estimates of SD or 2D which can differ from administrative coverage [31]. Indeed, one coverage survey data was available from 2D pre-emptive vaccination in 2022, implemented under ECCP project. The 2D coverage estimate was approximately 80% (78% in Shashemene Town; 83% in Shashemene Woreda) whereas administrative coverages of first and second round reached nearly 100% [13]. Coverage surveys including age and sex demographics provide important information regarding vaccine acceptance as well as reasons for non-vaccination which are important for improving future campaigns [32–36]. In addition, OCV is known to provide indirect or herd protection enhancing the overall impact, but which can vary with coverage rate [37, 38]. With the data available currently in Ethiopia, it is difficult to know actual coverage per dose or have data to inform improvement in future campaigns. Fortunately, as of November 2023, EPHI is conducting coverage studies in Afar, Oromia, South and Central Ethiopia regions to collect comprehensive coverage data using a pre-developed protocol. The coverage survey will be implemented within 2 weeks of campaign end date for every future OCV campaign.

## CONCLUSION

To our knowledge, this is the first comprehensive review paper documenting all OCV requests made by the Ethiopian government from 2019 to October 2023 and mass vaccination campaigns implemented across the country in the last 5 years. Five full approvals and six partial approvals were made by the ICG, and 66.3% of OCV requests were approved. Of the approved OCV doses, 90.4% were delivered to Ethiopia. In spite of many challenges, the Ethiopian government was able to conduct numerous campaigns both reactive and preemptive in many settings, achieving high administrative coverage. Because of the inherent inaccuracies in administrative coverage data, coverage surveys should be carried out in future campaigns. A comprehensive review of past OCV vaccinations implemented in Ethiopia not only supports better planning of effective national OCV vaccination in the future but also generates the framework for future research areas.

## Supplementary Data

Supplementary materials are available at *Clinical Infectious Diseases* online. Consisting of data provided by the authors to benefit the reader, the posted materials are not copyedited and are the sole responsibility of the authors, so questions or comments should be addressed to the corresponding author.

## Supplementary Material

ciae194_Supplementary_Data

## Notes

***Author contributions*.** Y. J., M. E., M. T., and S. E. P. conceptualized the overall manuscript. Database review, data analysis, and manuscript drafting were led by Y. J. with M. E. in discussion with S. E. P. and M. T. All authors read and provided feedback and approved the final draft.

***Acknowledgments***. The authors acknowledge the EPHI (M. E., Y. W., M. H., M. W.) for the access to the national health data system. We thank health professionals and local and regional government officials in each woreda and regional health bureau for their effort to supervise oral cholera vaccine (OCV) vaccination and collect the data.

***Data sharing*.** All data relevant to the study are included in this manuscript.

***Financial support***. This study was supported by the Korea Support Committee for International Vaccine Institute (CHMTD05083-010), LG Electronics (CHMTD05083-020), and Community Chest of Korea (CHMTD05083-030). The International Vaccine Institute (IVI) acknowledges its donors, including the government of the Republic of Korea and the Swedish International Development Cooperation Agency.

***Supplement sponsorship.*** This article appears as part of the supplement “Ethiopia Cholera Control and Prevention (ECCP): Evidence-Generation Towards Global Roadmap to Ending Cholera,” sponsored by the IVI.
