# Supplementary material for: Comprehensive Review on the Use of Oral Cholera Vaccine (OCV) in Ethiopia: 2019 to 2023
Source: Clin Infect Dis. 2024 Jul 12;79(Suppl 1):S20–32. doi: 10.1093/cid/ciae194 (PMC11244176; doi:10.1093/cid/ciae194)
Supplement: ciae194_Supplementary_Data [file ciae194_supplementary_data.docx]

**Supplementary Table 1. Detailed OCV vaccination campaigns in Ethiopia from 2019 to 2023**

| **Request Number^1^** | **Region/Zone^2^/Woreda(District)^3^** | | | **Total Population^4^** | **Population of targeted kebeles^5^** | **OCV Target Population^6^** | **Single dose (SD) or Two-doses (2D) OCV vaccination campaign** | **1^st^ Round** | | | | **Dose Interval**  **(if two doses administered)** | **2^nd^ Round** | | | |
| --- | --- | --- | --- | --- | --- | --- | --- | --- | --- | --- | --- | --- | --- | --- | --- | --- |
|  |  |  |  |  |  |  |  | **Campaign Start date**  **(YYYY/MM/DD)** | **Campaign End date (YYYY/MM/DD)** | **Number of people vaccinated with OCV(n)** | **Administrative Coverage^7^ (%)** |  | **Campaign Start date (YYYY/MM/DD)** | **Campaign End date (YYYY/MM/DD)** | **Number of people vaccinated with OCV (n)** | **Administrative Coverage (%)** |
| **Bilateral**^8^ | **SNNPR^9^** | | | | | | | | | | | | | | | |
|  |  | **Gamo** | | | | | | | | | | | | | | |
|  |  |  | Kamba | 135,620 | n.a^10^ | 12,200 | SD | 2019/07/01 | 2019/07/05 | 12,148 | 99.6 | n.a | n.a | n.a | n.a | n.a |
|  |  | **Goda** | | | | | | | | | | | | | | |
|  |  |  | Uba Debretsehay | 93,076 | n.a | 18,000 | SD | 2019/07/01 | 2019/07/05 | 17,820 | 99.0 | n.a | n.a | n.a | n.a | n.a |
|  |  | **Gofa** | | | | | | | | | | | | | | |
|  |  |  | Zala | 99,560 | n.a | 14,500 | SD | 2019/07/01 | 2019/07/05 | 14,065 | 97.0 | n.a | n.a | n.a | n.a | n.a |
|  |  | **South Omo** | | | | | | | | | | | | | | |
|  |  |  | Selemago | 37,391 | n.a | 26,300 | SD | 2019/07/01 | 2019/07/05 | 26,196 | 99.6 | n.a | n.a | n.a | n.a | n.a |
|  | **Total** | | | **365,647** | n.a | **71,000** | **SD** | 2019/07/01 | 2019/07/05 | **70,229** | **98.9** | n.a | n.a | n.a | n.a | n.a |
|  | **Somali** | | | | | | | | | | | | | | | |
|  |  | **Shebele** | | | | | | | | | | | | | | |
|  |  |  | Hudet | 108,554 | n.a | 32,500 | SD | 2019/07/01 | 2019/07/05 | 32,286 | 99.3 | n.a | n.a | n.a | n.a | n.a |
|  |  |  | Kelafo | 61,122 | n.a | 58,947 |  | 2019/07/01 | 2019/07/05 | 54,231 | 92.0 | n.a | n.a | n.a | n.a | n.a |
|  |  | **Siti** | | | | | | | | | | | | | | |
|  |  |  | Erer | 95,215 | n.a | 47,000 | SD | 2019/07/01 | 2019/07/05 | 46,040 | 98.0 | n.a | n.a | n.a | n.a | n.a |
|  | **Total** | | | **264,891** | n.a | **138,447** | **SD** | **2019/07/01** | **2019/07/05** | **132,557** | **95.7** | **n.a** | **n.a** | **n.a** | **n.a** | **n.a** |
| **Total-Bilateral** | | | | **630,538** | **n.a** | **209,447** | **SD** | **2019/07/01** | **2019/07/05** | **202,786** | **96.8** | **n.a** | **n.a** | **n.a** | **n.a** | **n.a** |
| **ECCP**^11^ | **Oromia** | | | | | | | | | | | | | | | |
|  |  | **West Arsi** | | | | | | | | | | | | | | |
|  |  |  | Shashemene Town | 291,589 | 163,546 | 40,250 | 2D | 2022/05/11 | 2022/05/16 | 41,056 | 102.0 | 2 weeks | 2022/05/27 | 2022/05/30 | 40,453 | 100.5 |
|  |  |  | Shashemene Woreda | 281,247 | 162,212 | 61,039 |  | 2022/05/11 | 2022/05/16 | 60,502 | 99.1 | 2 weeks | 2022/05/27 | 2022/06/01 | 60,480 | 100.0 |
| **Total-ECCP** | | | | **572,836** | **325,758** | **101,289** | **2D** | **2022/05/11** | **2022/05/16** | **101,558** | **100.3** | **2 weeks** | **2022/05/27** | **2022/06/01** | **100,933** | **99.6** |
| **Total-outside of global stockpile** | | | | **1,203,374** | **325,758** | **310,736** | **n.a** | **n.a** | **n.a** | **304,344** | **97.9** | **n.a** | **n.a** | **n.a** | **100,933** | **99.6** |
| **A1** | **Oromia** | | | | | | | | | | | | | | | |
|  |  | **West Hararge** | | | | | | | | | | | | | | |
|  |  |  | Bedessa | 47,468 | 28,655 | 27,824 | 2D | 2019/07/01 | 2019/07/05 | 27,136 | 97.5 | 24 weeks | 2019/12/14 | 2019/12/24 | 28,881 | 103.8 |
|  |  |  | Chiro | 237,169 | 104,034 | 101,017 |  | 2019/07/01 | 2019/07/05 | 98,532 | 97.5 | 24 weeks | 2019/12/14 | 2019/12/24 | 96,281 | 95.3 |
|  |  |  | Gemechise | 244,256 | 30,673 | 29,783 |  | 2019/07/01 | 2019/07/05 | 28,998 | 97.4 | 24 weeks | 2019/12/14 | 2019/12/24 | 55,762 | 187.2 |
|  |  |  | Mieso | 143,316 | 83,139 | 80,728 |  | 2019/07/01 | 2019/07/05 | 78,784 | 97.6 | 24 weeks | 2019/12/14 | 2019/12/24 | 79,337 | 98.3 |
|  |  |  | Oda Bultum | 220,936 | 60,753 | 58,991 |  | 2019/07/01 | 2019/07/05 | 57,290 | 97.1 | 24 weeks | 2019/12/14 | 2019/12/24 | 25,942 | 44.0 |
|  | **Total** | | | **893,145** | **307,254** | **298,343** | **2D** | **2019/07/01** | **2019/07/05** | **290,740** | **97.5** | **24 weeks** | **2019/12/14** | **2019/12/24** | **286,203** | **95.9** |
|  | **Addis Ababa** | | | | | | | | | | | | | | | |
|  |  | **Camps^12^** | | | | | | | | | | | | | | |
|  |  |  | Camps | 3,730,931 | 18,762 | 17,324 | SD | 2019/07/01 | 2019/07/05 | 17,324 | 100.0 | n.a | n.a | n.a | n.a | n.a |
|  | **Total** | | | **3,730,931** | **18,762** | **17,324** | **SD** | **2019/07/01** | **2019/07/05** | **17,324** | **100.0** | **n.a** | **n.a** | **n.a** | **n.a** | **n.a** |
|  | **Afar** | | | | | | | | | | | | | | | |
|  |  | **Zone 3** | | | | | | | | | | | | | | |
|  |  |  | Amibara | 77,878 | 64,492 | 62,622 | SD | 2019/07/01 | 2019/07/05 | 51,933 | 82.9 | n.a | n.a | n.a | n.a | n.a |
|  |  | **Zone 4** | | | | | | | | | | | | | | |
|  |  |  | Ewa | 58,481 | 38,829 | 29,448 | SD | 2019/07/01 | 2019/07/05 | 29,448 | 100.0 | n.a | n.a | n.a | n.a | n.a |
|  | **Total** | | | **136,359** | **103,321** | **92,070** | **SD** | **2019/07/01** | **2019/07/05** | **81,381** | **100.0** | **n.a** | **n.a** | **n.a** | **n.a** | **n.a** |
|  | **Sidama** | | | | | | | | | | | | | | | |
|  |  | **Hawassa** | | | | | | | | | | | | | | |
|  |  |  | Hawassa/IPDC^13^ | n.a | 35,015 | 23,322 | SD | 2019/07/01 | 2019/07/05 | 23,322 | 88.4 | n.a | n.a | n.a | n.a | n.a |
|  | **Total** | | | **n.a** | **35,015** | **23,322** | **SD** | **2019/07/01** | **2019/07/05** | **23,322** | **88.4** | **n.a** | **n.a** | **n.a** | **n.a** | **n.a** |
| **Total-A1** | | | | **4,760,435** | **464,352** | **431,059** | **SD/2D (Oromia)** | **2019/07/01** | **2019/07/05** | **412,767** | **95.8** | **24 weeks** | **2019/12/14** | **2019/12/24** | **286,203** | **95.9** |
| **A2** | **Gambella** | | | | | | | | | | | | | | | |
|  |  | **Agnuak** | | | | | | | | | | | | | | |
|  |  |  | Dima | 27,938 | 27,938 | 27,128 | 2D | 2020/12/21 | 2020/12/29 | 24,226 | 89.3 | 9 weeks | 2021/02/28 | 2021/03/06 | 24,226 | 89 |
|  | **Total** | | | **27,938** | **27,938** | **27,128** | **2D** | **2020/12/21** | **2020/12/29** | **24,226** | **89.3** | **9 weeks** | **2021/02/28** | **2021/03/06** | **24,226** | **89.3** |
|  | **Oromia** | | | | | | | | | | | | | | | |
|  |  | **Guji** | | | | | | | | | | | | | | |
|  |  |  | Adola Rede | 158,177 | 155,887 | 151,366 | 2D | 2020/12/21 | 2020/12/29 | 143,477 | 94.8 | 9 weeks | 2021/02/28 | 2021/03/06 | 137,876 | 91.1 |
|  |  |  | Liben | 97,456 | 45,291 | 43,978 |  | 2020/12/21 | 2020/12/29 | 35,703 | 81.2 | 9 weeks | 2021/02/28 | 2021/03/06 | 35,703 | 81.2 |
|  |  |  | Negele Town | 63,570 | 58,123 | 56,437 |  | 2020/12/21 | 2020/12/29 | 54,075 | 95.8 | 9 weeks | 2021/02/28 | 2021/03/06 | 54,075 | 95.8 |
|  |  | **West Arsi** | | | | | | | | | | | | | | |
|  |  |  | Nensebo | 63,570 | 32,887 | 31,933 | 2D | 2020/12/21 | 2020/12/29 | 32,745 | 102.5 | 9 weeks | 2021/02/28 | 2021/03/06 | 32,745 | 102.5 |
|  |  | **West Guji** | | | | | | | | | | | | | | |
|  |  |  | Abaya | 157,705 | 157,705 | 153,132 | 2D | 2020/12/21 | 2020/12/29 | 145,488 | 95.0 | 9 weeks | 2021/02/28 | 2021/03/06 | 149,849 | 97.9 |
|  |  |  | Gelana | 105,367 | 55,641 | 54,027 |  | 2020/12/21 | 2020/12/29 | 59,690 | 110.5 | 9 weeks | 2021/02/28 | 2021/03/06 | 59,690 | 110.5 |
|  |  |  | Melkasoda | 88,729 | 13,337 | 12,950 |  | 2020/12/21 | 2020/12/29 | 12,403 | 95.8 | 9 weeks | 2021/02/28 | 2021/03/06 | 12,403 | 95.8 |
|  | **Total** | | | **734,574** | **518,871** | **503,823** | **2D** | **2020/12/21** | **2020/12/29** | **483,581** | **96.0** | **9 weeks** | **2021/02/28** | **2021/03/06** | **482,341** | **95.7** |
|  | **Sidama** | | | | | | | | | | | | | | | |
|  |  | **Sidama** | | | | | | | | | | | | | | |
|  |  |  | Aroresa | 105,616 | 96,382 | 93,587 | 2D | 2020/12/21 | 2020/12/29 | 89,696 | 95.8 | 9 weeks | 2021/02/28 | 2021/03/06 | 92,289 | 98.6 |
|  |  |  | Bona Zuria | 154,541 | 135,662 | 131,728 |  | 2020/12/21 | 2020/12/29 | 135,319 | 102.7 | 9 weeks | 2021/02/28 | 2021/03/06 | 133,995 | 101.7 |
|  |  |  | Chabe Gambiltu | 82,269 | 48,640 | 47,229 |  | 2020/12/21 | 2020/12/29 | 45,178 | 95.7 | 9 weeks | 2021/02/28 | 2021/03/06 | 43,725 | 92.6 |
|  |  |  | Huko | 98,505 | 89,219 | 86,632 |  | 2020/12/21 | 2020/12/29 | 81,996 | 94.6 | 9 weeks | 2021/02/28 | 2021/03/06 | 81,836 | 94.5 |
|  | **Total** | | | **440,931** | **369,903** | **359,176** | **2D** | **2020/12/21** | **2020/12/29** | **352,189** | **98.1** | **9 weeks** | **2021/02/28** | **2021/03/06** | **351,845** | **98.0** |
|  | **SNNPR** | | | | | | | | | | | | | | | |
|  |  | **Dawuro** | | | | | | | | | | | | | | |
|  |  |  | Gena | 45,660 | 45,660 | 44,336 | 2D | 2020/12/21 | 2020/12/29 | 39,982 | 90.2 | 9 weeks | 2021/02/28 | 2021/03/06 | 39,982 | 90.2 |
|  |  |  | Tercha Town | 25,605 | 25,605 | 24,862 |  | 2020/12/21 | 2020/12/29 | 22,880 | 92.0 | 9 weeks | 2021/02/28 | 2021/03/06 | 22,880 | 92.0 |
|  |  |  | Tercha Zuria | 70,287 | 70,287 | 68,249 |  | 2020/12/21 | 2020/12/29 | 54,560 | 79.9 | 9 weeks | 2021/02/28 | 2021/03/06 | 53,099 | 77.8 |
|  |  | **South Omo** | | | | | | | | | | | | | | |
|  |  |  | Benatsemay | 73,170 | 34,963 | 33,949 | 2D | 2020/12/21 | 2020/12/29 | 29,752 | 87.6 | 9 weeks | 2021/02/28 | 2021/03/06 | 24,207 | 71.3 |
|  |  |  | Dasenech | 73,402 | 73,402 | 71,273 |  | 2020/12/21 | 2020/12/29 | 69,675 | 97.8 | 9 weeks | 2021/02/28 | 2021/03/06 | 66,974 | 94.0 |
|  |  |  | Hamer | 82,475 | 41,970 | 40,753 |  | 2020/12/21 | 2020/12/29 | 38,034 | 93.3 | 9 weeks | 2021/02/28 | 2021/03/06 | 33,476 | 82.1 |
|  |  |  | Mallei | 116,417 | 77,419 | 75,174 |  | 2020/12/21 | 2020/12/29 | 74,164 | 98.7 | 9 weeks | 2021/02/28 | 2021/03/06 | 66,532 | 88.5 |
|  |  |  | Ngangatom | 24,203 | 11,714 | 11,374 |  | 2020/12/21 | 2020/12/29 | 10,929 | 96.1 | 9 weeks | 2021/02/28 | 2021/03/06 | 10,929 | 96.1 |
|  |  | **West Omo** | | | | | | | | | | | | | | |
|  |  |  | Bero | 21,560 | 21,560 | 20,935 | 2D | 2020/12/21 | 2020/12/29 | 19,706 | 94.1 | 9 weeks | 2021/02/28 | 2021/03/06 | 19,318 | 92.3 |
|  |  |  | Gachit | 64,439 | 64,439 | 62,570 |  | 2020/12/21 | 2020/12/29 | 57,308 | 91.6 | 9 weeks | 2021/02/28 | 2021/03/06 | 55,077 | 88.0 |
|  |  |  | Menit Goldiya | 77,377 | 77,377 | 75,133 |  | 2020/12/21 | 2020/12/29 | 75,822 | 100.9 | 9 weeks | 2021/02/28 | 2021/03/06 | 75,822 | 100.9 |
|  |  |  | Menit Shasha | 37,464 | 37,464 | 36,378 |  | 2020/12/21 | 2020/12/29 | 35,877 | 98.6 | 9 weeks | 2021/02/28 | 2021/03/06 | 35,877 | 98.6 |
|  |  |  | Surma | 34,207 | 34,207 | 33,215 |  | 2020/12/21 | 2020/12/29 | 30,916 | 93.1 | 9 weeks | 2021/02/28 | 2021/03/06 | 30,026 | 90.4 |
|  | **Total** | | | **746,266** | **616,067** | **598,201** | **2D** | **2020/12/21** | **2020/12/29** | **559,605** | **93.5** | **9 weeks** | **2021/02/28** | **2021/03/06** | **534,199** | **89.3** |
|  | **Somali** | | | | | | | | | | | | | | | |
|  |  | **Shebele** | | | | | | | | | | | | | | |
|  |  |  | Fer Fer | 56,721 | 23,808 | 23,118 | 2D | 2020/12/21 | 2020/12/29 | 24,621 | 106.5 | 9 weeks | 2021/02/28 | 2021/03/06 | 24,677 | 106.7 |
|  |  |  | Kelafo | 111,452 | 43,511 | 42,249 |  | 2020/12/21 | 2020/12/29 | 45,686 | 108.1 | 9 weeks | 2021/02/28 | 2021/03/06 | 45,506 | 107.7 |
|  |  |  | Mustahkin | 67,937 | 50,076 | 48,624 |  | 2020/12/21 | 2020/12/29 | 54,556 | 112.2 | 9 weeks | 2021/02/28 | 2021/03/06 | 52,420 | 107.8 |
|  | **Total** | | | **236,110** | **117,395** | **113,991** | **2D** | **2020/12/21** | **2020/12/29** | **124,863** | **109.5** | **9 weeks** | **2021/02/28** | **2021/03/06** | **122,603** | **107.6** |
| **Total-A2** | | | | **2,185,819^14^** | **1,650,174** | **1,602,319** | **2D** | **2020/12/21** | **2020/12/29** | **1,544,464** | **96.4** | **9 weeks** | **2021/02/28** | **2021/03/06** | **1,515,214** | **94.6** |
| **A3** | **Oromia** | | | | | | | | | | | | | | | |
|  |  | **West Arsi** | | | | | | | | | | | | | | |
|  |  |  | Nensebo | 155,743 | 155,743 | 151,227 | 2D | 2021/05/20 | 2021/05/26 | 148,201 | 98.0 | 12 weeks | 2021/08/18 | 2021/08/25 | 148,201 | 100.0 |
|  | **Total** | | | **155,743** | **155,743** | **151,227** | **2D** | **2021/05/20** | **2021/05/26** | **148,201** | **98.0** | **12 weeks** | **2021/08/18** | **2021/08/25** | **148,201** | **100.0** |
|  | **SNNPR** | | | | | | | | | | | | | | | |
|  |  | **Bench Sheko Zone** | | | | | | | | | | | | | | |
|  |  |  | Mizan Aman | 74,348 | 74,348 | 72,192 | 2D | 2021/05/20 | 2021/05/26 | 71,758 | 99.4 | 12 weeks | 2021/08/18 | 2021/08/25 | 71,758 | 100.0 |
|  |  |  | Semen Bench | 103,539 | 103,539 | 100,537 |  | 2021/05/20 | 2021/05/26 | 92,696 | 92.2 | 12 weeks | 2021/08/18 | 2021/08/25 | 92,696 | 100.0 |
|  |  |  | Shebench | 150,273 | 150,273 | 145,916 |  | 2021/05/20 | 2021/05/26 | 145,818 | 99.9 | 12 weeks | 2021/08/18 | 2021/08/25 | 145,118 | 99.5 |
|  |  |  | Sheko | 70,865 | 70,865 | 68,810 |  | 2021/05/20 | 2021/05/26 | 65,507 | 95.2 | 12 weeks | 2021/08/18 | 2021/08/25 | 64,515 | 98.5 |
|  |  |  | Siz Town | 47,224 | 47,224 | 45,855 |  | 2021/05/20 | 2021/05/26 | 45,815 | 99.9 | 12 weeks | 2021/08/18 | 2021/08/25 | 45,815 | 100.0 |
|  |  |  | South Bench | 153,078 | 153,078 | 148,639 |  | 2021/05/20 | 2021/05/26 | 148,044 | 99.6 | 12 weeks | 2021/08/18 | 2021/08/25 | 148,044 | 100.0 |
|  |  | **Dawro** | | | | | | | | | | | | | | |
|  |  |  | Gena | 46,083 | 46,083 | 44,747 | 2D | 2021/05/20 | 2021/05/26 | 44,120 | 98.6 | 12 weeks | 2021/08/18 | 2021/08/25 | 44,019 | 99.8 |
|  |  |  | Isara | 91,542 | 91,542 | 88,888 |  | 2021/05/20 | 2021/05/26 | 83,356 | 93.8 | 12 weeks | 2021/08/18 | 2021/08/25 | 83,314 | 99.9 |
|  |  |  | Kechi | 30,979 | 30,979 | 30,081 |  | 2021/05/20 | 2021/05/26 | 29,966 | 99.6 | 12 weeks | 2021/08/18 | 2021/08/25 | 29,902 | 99.8 |
|  |  |  | Mari Mansa | 78,246 | 78,246 | 75,977 |  | 2021/05/20 | 2021/05/26 | 74,555 | 98.1 | 12 weeks | 2021/08/18 | 2021/08/25 | 73,556 | 98.7 |
|  |  |  | Tercha Town admin | 25,842 | 25,842 | 25,093 |  | 2021/05/20 | 2021/05/26 | 24,892 | 99.2 | 12 weeks | 2021/08/18 | 2021/08/25 | 24,892 | 100.0 |
|  |  |  | Tercha Zuriya | 70,938 | 70,938 | 68,881 |  | 2021/05/20 | 2021/05/26 | 67,641 | 98.2 | 12 weeks | 2021/08/18 | 2021/08/25 | 67,641 | 100.0 |
|  |  |  | Tocha | 132,218 | 132,218 | 128,384 |  | 2021/05/20 | 2021/05/26 | 125,570 | 97.8 | 12 weeks | 2021/08/18 | 2021/08/25 | 125,168 | 99.7 |
|  | **Total** | | | **1,075,175** | **1,075,175** | **1,044,000** | **2D** | **2021/05/20** | **2021/05/26** | **1,019,738** | **97.7** | **12 weeks** | **2021/08/18** | **2021/08/25** | **1,016,438** | **99.7** |
| **Total-A3** | | | | **1,230,918** | **1,230,918** | **1,195,227** | **2D** | **2021/05/20** | **2021/05/26** | **1,167,939** | **97.7** | **12 weeks** | **2021/08/18** | **2021/08/25** | **1,164,639** | **99.7** |
| **A4** | **Tigray^15^** | | | | | | | | | | | | | | | |
|  |  | **Central** | | | | | | | | | | | | | | |
|  |  |  | AbiAdi Town | 34,572 | 72,004 | 69,916 | SD | 2021/06/10 | 2021/06/17 | 25,780 | 36.9 | n.a | n.a | n.a | n.a | n.a |
|  |  |  | Adwa Town | 84,227 | 220,730 | 214,329 |  | 2021/06/10 | 2021/06/17 | 70,762 | 33.0 | n.a | n.a | n.a | n.a | n.a |
|  |  |  | Axum Town | 81,065 | 151,872 | 147,468 |  | 2021/06/10 | 2021/06/17 | 74,101 | 50.2 | n.a | n.a | n.a | n.a | n.a |
|  |  | **Eastern** | | | | | | | | | | | | | | |
|  |  |  | Adigrat Town | 81,065 | 215,855 | 209,595 | SD | 2021/06/10 | 2021/06/17 | 101,242 | 48.3 | n.a | n.a | n.a | n.a | n.a |
|  |  | **Mekelle** | | | | | | | | | | | | | | |
|  |  |  | Mekelle | 356,845 | 188,233 | 182,774 | SD | 2021/06/10 | 2021/06/17 | 97,390 | 53.3 | n.a | n.a | n.a | n.a | n.a |
|  |  | **North Western** | | | | | | | | | | | | | | |
|  |  |  | Asgede | 92,822 | 137,303 | 133,321 | SD | 2021/06/10 | 2021/06/17 | 26,543 | 19.9 | n.a | n.a | n.a | n.a | n.a |
|  |  |  | Sheraro Town | 30,984 | 88,548 | 85,980 |  | 2021/06/10 | 2021/06/17 | 20,109 | 23.4 | n.a | n.a | n.a | n.a | n.a |
|  |  |  | Shire Town | 91,537 | 442,508 | 429,675 |  | 2021/06/10 | 2021/06/17 | 121,487 | 28.3 | n.a | n.a | n.a | n.a | n.a |
|  |  |  | Tahtay Koraro | 89,883 | 126,547 | 122,877 |  | 2021/06/10 | 2021/06/17 | 55,412 | 45.1 | n.a | n.a | n.a | n.a | n.a |
|  |  | **South Eastern** | | | | | | | | | | | | | | |
|  |  |  | Degua Temben | 71,910 | 73,245 | 71,121 | SD | 2021/06/10 | 2021/06/17 | 33,440 | 47.0 | n.a | n.a | n.a | n.a | n.a |
|  |  |  | Hintalo | 111,277 | 152,596 | 148,171 |  | 2021/06/10 | 2021/06/17 | 93,788 | 63.3 | n.a | n.a | n.a | n.a | n.a |
|  |  |  | Saharti | 87,889 | 121,283 | 117,766 |  | 2021/06/10 | 2021/06/17 | 85,878 | 72.9 | n.a | n.a | n.a | n.a | n.a |
|  |  | **Southern** | | | | | | | | | | | | | | |
|  |  |  | Maichew Town | 42,485 | 80,007 | 77,687 | SD | 2021/06/10 | 2021/06/17 | 34,842 | 44.8 | n.a | n.a | n.a | n.a | n.a |
| **Total-A4** | | | | **1,256,561** | **2,070,731** | **2,010,680** | **SD** | **2021/06/10** | **2021/06/17** | **840,774** | **41.8** | **n.a** | **n.a** | **n.a** | **n.a** | **n.a** |
| **A5** | **Oromia** | | | | | | | | | | | | | | | |
|  |  | **Bale** | | | | | | | | | | | | | | |
|  |  |  | Berbere | 133,308 | 133,308 | 130,642 | 2D | 2021/12/23 | 2022/01/01 | 130,201 | 99.7 | 13 weeks | 2022/03/25 | 2022/03/31 | 130,201 | 99.7 |
|  |  |  | D/menaa | 133,213 | 133,213 | 130,549 |  | 2021/12/23 | 2022/01/01 | 130,130,532 | 100.0 | 13 weeks | 2022/03/25 | 2022/03/31 | 130,542 | 100.0 |
|  |  |  | H/Buluk | 119,809 | 119,809 | 117,413 |  | 2021/12/23 | 2022/01/01 | 117,349 | 99.9 | 13 weeks | 2022/03/25 | 2022/03/31 | 117,345 | 99.9 |
|  |  |  | Meda Welabu | 142,628 | 142,628 | 139,775 |  | 2021/12/23 | 2022/01/01 | 138,752 | 99.3 | 13 weeks | 2022/03/25 | 2022/03/31 | 138,746 | 99.3 |
|  | **Total** | | | **528,958** | **528,958** | **518,379** | **2D** | **2021/12/23** | **2022/01/01** | **516,834** | **99.7** | **13 weeks** | **2022/03/25** | **2022/03/31** | **516,834** | **99.7** |
|  | **Somali** | | | | | | | | | | | | | | | |
|  |  | **Liban** | | | | | | | | | | | | | | |
|  |  |  | Bokolmayo | 52,136 | 52,136 | 51,093 | 2D | 2021/12/23 | 2022/01/01 | 51,093 | 100.0 | 13 weeks | 2022/03/25 | 2022/03/31 | 51,093 | 100.0 |
|  |  |  | Deka Suftu | 60,224 | 60,224 | 59,020 |  | 2021/12/23 | 2022/01/01 | 58,989 | 99.9 | 13 weeks | 2022/03/25 | 2022/03/31 | 58,987 | 99.9 |
|  |  |  | Dolo Odo | 145,050 | 145,050 | 142,149 |  | 2021/12/23 | 2022/01/01 | 142,068 | 99.9 | 13 weeks | 2022/03/25 | 2022/03/31 | 142,064 | 99.9 |
|  |  |  | Filtu | 114,980 | 114,980 | 112,680 |  | 2021/12/23 | 2022/01/01 | 112,597 | 99.9 | 13 weeks | 2022/03/25 | 2022/03/31 | 112,593 | 99.9 |
|  |  |  | Goro Baqaqsa | 68,612 | 68,612 | 67,240 |  | 2021/12/23 | 2022/01/01 | 67,149 | 99.9 | 13 weeks | 2022/03/25 | 2022/03/31 | 67,148 | 99.9 |
|  |  |  | Gura Damole | 27,984 | 27,984 | 27,424 |  | 2021/12/23 | 2022/01/01 | 27,424 | 100.0 | 13 weeks | 2022/03/25 | 2022/03/31 | 27,424 | 100.0 |
|  |  |  | Kersadula | 62,239 | 62,239 | 60,994 |  | 2021/12/23 | 2022/01/01 | 60,715 | 99.5 | 13 weeks | 2022/03/25 | 2022/03/31 | 60,716 | 99.5 |
|  | **Total** | | | **531,225** | **531,225** | **520,600** | **531,225** | **2021/12/23** | **2022/01/01** | **520,035** | **99.9** | **13 weeks** | **2022/03/25** | **2022/03/31** | **520,025** | **99.9** |
| **Total-A5** | | | | **1,060,183^16^** | **1,060,183** | **1,038,979** | **2D** | **2021/12/23** | **2022/01/01** | **1,036,869** | **99.8** | **13 weeks** | **2022/03/25** | **2022/03/31** | **1,036,859** | **99.8** |
| **N/A^17^** | **Afar** | | | | | | | | | | | | | | | |
|  |  | **Zone 1** | | | | | | | | | | | | | | |
|  |  |  | Adaar | 70,540 | 13,186 | 13,186 | SD | 2022/04/23 | 2022/04/30 | 12,886 | 98 | n.a | n.a | n.a | n.a | n.a |
|  |  |  | Chifra | 119,417 | 15,392 | 15,392 |  | 2022/04/23 | 2022/04/30 | 15,192 | 99 | n.a | n.a | n.a | n.a | n.a |
|  |  |  | Kori | 63,628 | 2,230 | 2,230 |  | 2022/04/23 | 2022/04/30 | 2,130 | 96 | n.a | n.a | n.a | n.a | n.a |
|  |  | **Zone 2** | | | | | | | | | | | | | | |
|  |  |  | Abaala | 49,749 | 10,708 | 10,708 | SD | 2022/04/23 | 2022/04/30 | 10,647 | 99 | n.a | n.a | n.a | n.a | n.a |
|  |  |  | Berahle | 104,062 | 6,220 | 6,220 |  | 2022/04/23 | 2022/04/30 | 6,120 | 98 | n.a | n.a | n.a | n.a | n.a |
|  |  |  | Erebti | 75,882 | 4,562 | 4,562 |  | 2022/04/23 | 2022/04/30 | 4,505 | 99 | n.a | n.a | n.a | n.a | n.a |
|  |  |  | Koneba | 71,021 | 7,294 | 7,294 |  | 2022/04/23 | 2022/04/30 | 7,244 | 99 | n.a | n.a | n.a | n.a | n.a |
|  |  |  | Megale | 36,851 | 9,883 | 9,883 |  | 2022/04/23 | 2022/04/30 | 9,813 | 99 | n.a | n.a | n.a | n.a | n.a |
|  |  | **Zone 3** | | | | | | | | | | | | | | |
|  |  |  | Afdera | 42,236 | 2,892 | 2,892 | SD | 2022/04/23 | 2022/04/30 | 2,738 | 95 | n.a | n.a | n.a | n.a | n.a |
|  |  | **Zone 4** | | | | | | | | | | | | | | |
|  |  |  | Awra | 46,881 | 12,270 | 12,270 | SD | 2022/04/23 | 2022/04/30 | 12,070 | 98 | n.a | n.a | n.a | n.a | n.a |
|  |  |  | Ewa | 62,487 | 12,012 | 12,012 |  | 2022/04/23 | 2022/04/30 | 12,012 | 100 | n.a | n.a | n.a | n.a | n.a |
|  |  |  | Golina | 65,283 | 12,635 | 12,635 |  | 2022/04/23 | 2022/04/30 | 12,535 | 99 | n.a | n.a | n.a | n.a | n.a |
|  |  |  | Teru | 62,226 | 13,678 | 13,678 |  | 2022/04/23 | 2022/04/30 | 13,529 | 99 | n.a | n.a | n.a | n.a | n.a |
|  |  |  | Yalo | 62,226 | 6,129 | 6,129 |  | 2022/04/23 | 2022/04/30 | 5,966 | 97 | n.a | n.a | n.a | n.a | n.a |
|  |  | **Zone 5** | | | | | | | | | | | | | | |
|  |  |  | Dewe | 55,492 | 8,283 | 8,283 | SD | 2022/04/23 | 2022/04/30 | 8,083 | 98 | n.a | n.a | n.a | n.a | n.a |
|  |  |  | Hadalela | 46,223 | 9,788 | 9,788 |  | 2022/04/23 | 2022/04/30 | 9,288 | 95 | n.a | n.a | n.a | n.a | n.a |
|  |  |  | Samurobi | 41,975 | 7,964 | 7,964 |  | 2022/04/23 | 2022/04/30 | 7,664 | 96 | n.a | n.a | n.a | n.a | n.a |
|  |  |  | Telalak | 49,771 | 8,418 | 8,418 |  | 2022/04/23 | 2022/04/30 | 7,918 | 94 | n.a | n.a | n.a | n.a | n.a |
|  | **Total** | | | **1,125,960** | **163,544** | **163,544** | **SD** | **2022/04/23** | **2022/04/30** | **160,340** | **98.0** | **n.a** | **n.a** | **n.a** | **n.a** | **n.a** |
|  | **Amhara** | | | | | | | | | | | | | | | |
|  |  | **Waghimra** | | | | | | | | | | | | | | |
|  |  |  | Dehana | 146,915 | 140,000 | 66,297 | SD | 2022/05/23 | 2022/05/31 | 64,343 | 97.1 | n.a | n.a | n.a | n.a | n.a |
|  |  |  | Gazgibla | 90,037 | 89,000 | 21,268 |  | 2022/05/23 | 2022/05/31 | 20,653 | 97.1 | n.a | n.a | n.a | n.a | n.a |
|  |  |  | Sahala | 33,986 | 33,000 | 17,990 |  | 2022/05/23 | 2022/05/31 | 17,128 | 95.2 | n.a | n.a | n.a | n.a | n.a |
|  |  |  | Sekota Town | 47,620 | 46,000 | 45,324 |  | 2022/05/23 | 2022/05/31 | 45,322 | 100.0 | n.a | n.a | n.a | n.a | n.a |
|  |  |  | Sekota Town (IDP^18^) | n.a | 43,605 | 25,231 |  | 2022/05/23 | 2022/05/31 | 13,723 | 54.4 | n.a | n.a | n.a | n.a | n.a |
|  |  |  | Sekota Zuria | 106,000 | 106,000 | 69,817 |  | 2022/05/23 | 2022/05/31 | 68,221 | 97.7 | n.a | n.a | n.a | n.a | n.a |
|  |  |  | Sekota Zuria (IDP) | n.a | 3,775 | 3,775 |  | 2022/05/23 | 2022/05/31 | 2,961 | 78.4 | n.a | n.a | n.a | n.a | n.a |
|  |  |  | Ziqwala | 62,029 | 41,892 | 41,892 |  | 2022/05/23 | 2022/05/31 | 32,837 | 78.4 | n.a | n.a | n.a | n.a | n.a |
|  | **Total** | | | **486,587** | **503,272** | **291,594** | **SD** | **2022/05/23** | **2022/05/31** | **265,188** | **90.9** | **n.a** | **n.a** | **n.a** | **n.a** | **n.a** |
| **Total – vaccination using retrieved vaccines** | | | | **1,612,547** | **666,816** | **455,138** | **SD** | **n.a** | **n.a** | **425,528** | **93.5** | **n.a** | **n.a** | **n.a** | **n.a** | **n.a** |
| **A6** | **Oromia** | | | | | | | | | | | | | | | |
|  |  | **Bale** | | | | | | | | | | | | | | |
|  |  |  | Berbere^19^ | 144,337 | 22,039 | 21,400 | SD | 2023/01/13 | 2023/01/20 | 21,351 | 99.8 | n.a | n.a | n.a | n.a | n.a |
|  |  |  | Goro | 133,671 | 56,643 | 55,000 |  | 2023/01/13 | 2023/01/20 | 54,875 | 99.8 | n.a | n.a | n.a | n.a | n.a |
|  | **Total** | | | **278,008** | **78,682** | **76,400** | **SD** | **2023/01/13** | **2023/01/20** | **76,226** | **99.8** | **n.a** | **n.a** | **n.a** | **n.a** | **n.a** |
|  | **Somali** | | | | | | | | | | | | | | | |
|  |  | **Liban** | | | | | | | | | | | | | | |
|  |  |  | Bokolmayo (IDP) | 39,181 | 8,441 | 8,196 | SD | 2023/01/13 | 2023/01/20 | 8,196 | 100.0 | n.a | n.a | n.a | n.a | n.a |
|  |  |  | Dolo Ado | 126,867 | 16,778 | 16,291 |  | 2023/01/13 | 2023/01/20 | 16,291 | 100.0 | n.a | n.a | n.a | n.a | n.a |
|  | **Total** | | | **166,048** | **25,219** | **24,487** | **SD** | **2023/01/13** | **2023/01/20** | **24,487** | **100** | **n.a** | **n.a** | **n.a** | **n.a** | **n.a** |
| **Total-A6** | | | | **444,056** | **103,901** | **100,887** | **SD** | **2023/01/13** | **2023/01/20** | **100,713** | **99.8** | **n.a** | **n.a** | **n.a** | **n.a** | **n.a** |
| **A7** | **Oromia** | | | | | | | | | | | | | | | |
|  |  | **Bale** | | | | | | | | | | | | | | |
|  |  |  | Berbere | 144,337 | 129,115 | 126,533 | SD | 2023/05/15 | 2023/05/24 | 126,331 | 99.8 | n.a | n.a | n.a | n.a | n.a |
|  |  |  | Goro | 133,671 | 116,031 | 113,710 |  | 2023/05/15 | 2023/05/24 | 113,589 | 99.9 | n.a | n.a | n.a | n.a | n.a |
|  |  |  | Gura Damole | 45,904 | 41,090 | 40,268 |  | 2023/05/15 | 2023/05/24 | 40,225 | 99.9 | n.a | n.a | n.a | n.a | n.a |
|  |  | **Borana** | | | | | | | | | | | | | | |
|  |  |  | Moyale | 311,469 | 311,469 | 305,240 | SD | 2023/05/15 | 2023/05/24 | 305,157 | 100.0 | n.a | n.a | n.a | n.a | n.a |
|  |  | **East Bale** | | | | | | | | | | | | | | |
|  |  |  | Dawe Kachen | 47,250 | 43,568 | 42,697 | SD | 2023/05/15 | 2023/05/24 | 42,677 | 100.0 | n.a | n.a | n.a | n.a | n.a |
|  |  |  | Ginir | 193,344 | 172,959 | 169,500 |  | 2023/05/15 | 2023/05/24 | 169,467 | 100.0 | n.a | n.a | n.a | n.a | n.a |
|  |  |  | Ginir Town | 55,379 | 55,379 | 54,271 |  | 2023/05/15 | 2023/05/24 | 54,195 | 99.9 | n.a | n.a | n.a | n.a | n.a |
|  |  |  | Raitu | 51,973 | 49,943 | 48,944 |  | 2023/05/15 | 2023/05/24 | 48,892 | 99.9 | n.a | n.a | n.a | n.a | n.a |
|  |  | **Guji** | | | | | | | | | | | | | | |
|  |  |  | Girja | 79,048 | 70,822 | 69,406 | SD | 2023/05/15 | 2023/05/24 | 69,394 | 100.0 | n.a | n.a | n.a | n.a | n.a |
|  |  |  | Goro Dola | 94,291 | 79,720 | 78,126 |  | 2023/05/15 | 2023/05/24 | 78,065 | 99.9 | n.a | n.a | n.a | n.a | n.a |
|  |  |  | Liben | 106,273 | 100,307 | 98,301 |  | 2023/05/15 | 2023/05/24 | 98,266 | 100.0 | n.a | n.a | n.a | n.a | n.a |
|  |  |  | Negele Town | 69,321 | 60,814 | 59,598 |  | 2023/05/15 | 2023/05/24 | 59,552 | 99.9 | n.a | n.a | n.a | n.a | n.a |
|  |  |  | Wadera | 81,129 | 68,592 | 67,220 |  | 2023/05/15 | 2023/05/24 | 67,163 | 99.9 | n.a | n.a | n.a | n.a | n.a |
|  |  | **West Arsi** | | | | | | | | | | | | | | |
|  |  |  | Nensebo | 197,753 | 197,753 | 193,798 | SD | 2023/05/15 | 2023/05/24 | 193,725 | 100.0 | n.a | n.a | n.a | n.a | n.a |
|  | **Total** | | | **1,611,142** | **1,497,562** | **1,467,612** | **SD** | **2023/05/15** | **2023/05/24** | **1,466,698** | **99.9** | **n.a** | **n.a** | **n.a** | **n.a** | **n.a** |
|  | **Somali** | | | | | | | | | | | | | | | |
|  |  | **Dawa** | | | | | | | | | | | | | | |
|  |  |  | Moyale | 279,530 | 271,709 | 266,275 | SD | 2023/05/15 | 2023/05/24 | 266,229 | 100.0 | n.a | n.a | n.a | n.a | n.a |
|  |  | **Liban** | | | | | | | | | | | | | | |
|  |  |  | Dollo Ado | 126,867 | 123,318 | 120,852 | SD | 2023/05/15 | 2023/05/24 | 120,804 | 100.0 | n.a | n.a | n.a | n.a | n.a |
|  |  |  | Guradamole | 56,815 | 56,815 | 55,679 |  | 2023/05/15 | 2023/05/24 | 55,674 | 100.0 | n.a | n.a | n.a | n.a | n.a |
|  | **Total** | | | **463,212** | **451,842** | **442,806** | **SD** | **2023/05/15** | **2023/05/24** | **442,707** | **100.0** | **n.a** | **n.a** | **n.a** | **n.a** | **n.a** |
| **Total-A7** | | | | **2,074,354** | **1,949,404** | **1,910,418** | **SD** | **2023/05/15** | **2023/05/24** | **1,909,405** | **99.9** | **n.a** | **n.a** | **n.a** | **n.a** | **n.a** |
| **A8** | **Oromia** | | | | | | | | | | | | | | | |
|  |  | **West Guji** | | | | | | | | | | | | | | |
|  |  |  | Bule Hora | 147,810 | 292,701 | 286,847 | SD | 2023/08/10 | 2023/08/20 | 286,802 | 100.0 | n.a | n.a | n.a | n.a | n.a |
|  |  |  | Dugda Dawa | 136,020 | 101,382 | 99,354 |  | 2023/08/10 | 2023/08/20 | 99,320 | 100.0 | n.a | n.a | n.a | n.a | n.a |
|  |  |  | Melka Soda | 96,756 | 86,258 | 84,533 |  | 2023/08/10 | 2023/08/20 | 84,456 | 99.9 | n.a | n.a | n.a | n.a | n.a |
|  |  |  | Liben Chukala | 120,852 | 102,577 | 100,525 |  | 2023/08/10 | 2023/08/20 | 100,524 | 100.0 | n.a | n.a | n.a | n.a | n.a |
|  | **Total** | | | **501,438** | **582,918** | **571,259** | **SD** | **2023/08/10** | **2023/08/20** | **571,102** | **100.0** | n.a | n.a | n.a | n.a | n.a |
|  | **Sidama** | | | | | | | | | | | | | | | |
|  |  | **Hawassa** | | | | | | | | | | | | | | |
|  |  |  | Hawassa Zuria | 186,240 | 71,712 | 70,278 | SD | 2023/08/10 | 2023/08/20 | 70,277 | 100.0 | n.a | n.a | n.a | n.a | n.a |
|  |  |  | Hawela | 116,048 | 151,259 | 148,234 |  | 2023/08/10 | 2023/08/20 | 148,234 | 100.0 | n.a | n.a | n.a | n.a | n.a |
|  |  | **Sidama** | | | | | | | | | | | | | | |
|  |  |  | Dara | 132,404 | 127,190 | 124,646 | SD | 2023/08/10 | 2023/08/20 | 124,645 | 100.0 | n.a | n.a | n.a | n.a | n.a |
|  | **Total** | | | **434,692** | **350,161** | **343,158** | **SD** | **2023/08/10** | **2023/08/20** | **343,156** | **100.0** | **n.a** | **n.a** | **n.a** | **n.a** | **n.a** |
|  | **SNNPR** | | | | | | | | | | | | | | | |
|  |  | **Alle Special** | | | | | | | | | | | | | | |
|  |  |  | Alle Special | 94,040 | 90,336 | 88,527 | SD | 2023/08/10 | 2023/08/20 | 88,527 | 100.0 | n.a | n.a | n.a | n.a | n.a |
|  |  | **Amaro Special** | | | | | | | | | | | | | | |
|  |  |  | Amaro | 218,912 | 165,268 | 161,963 | SD | 2023/08/10 | 2023/08/20 | 161,961 | 100.0 | n.a | n.a | n.a | n.a | n.a |
|  |  | **Burji Special** | | | | | | | | | | | | | | |
|  |  |  | Burji Special | 85,231 | 81,875 | 80,238 | SD | 2023/08/10 | 2023/08/20 | 80,235 | 100.0 | n.a | n.a | n.a | n.a | n.a |
|  |  | **Gamo** | | | | | | | | | | | | | | |
|  |  |  | Arba Minch Zuria | 137,296 | 256,889 | 251,751 | SD | 2023/08/10 | 2023/08/20 | 251,749 | 100.0 | n.a | n.a | n.a | n.a | n.a |
|  |  | **Gedeo** | | | | | | | | | | | | | | |
|  |  |  | Sawla Town | 51,854 | 49,328 | 48,331 | SD | 2023/08/10 | 2023/08/20 | 48,289 | 99.9 | n.a | n.a | n.a | n.a | n.a |
|  |  | **Gofa** | | | | | | | | | | | | | | |
|  |  |  | Denba Gofa | 109,249 | 104,947 | 102,848 | SD | 2023/08/10 | 2023/08/20 | 102,842 | 100.0 | n.a | n.a | n.a | n.a | n.a |
|  |  | **Hadiya** | | | | | | | | | | | | | | |
|  |  |  | Gerese | 172,903 | 130,725 | 128,111 | SD | 2023/08/10 | 2023/08/20 | 128,111 | 100.0 | n.a | n.a | n.a | n.a | n.a |
|  |  | **Konso** | | | | | | | | | | | | | | |
|  |  |  | Karat Zuria | 89,752 | 134,649 | 131,956 | SD | 2023/08/10 | 2023/08/20 | 131,938 | 100.0 | n.a | n.a | n.a | n.a | n.a |
|  |  |  | Kena | 86,845 | 80,685 | 79,071 |  | 2023/08/10 | 2023/08/20 | 79,070 | 100.0 | n.a | n.a | n.a | n.a | n.a |
|  |  |  | Segen Zuria | 52,231 | 49,328 | 48,341 |  | 2023/08/10 | 2023/08/20 | 48,338 | 100.0 | n.a | n.a | n.a | n.a | n.a |
|  |  | **South Omo** | | | | | | | | | | | | | | |
|  |  |  | Bena Tsemay | 79,789 | 76,647 | 75,114 | SD | 2023/08/10 | 2023/08/20 | 75,113 | 100.0 | n.a | n.a | n.a | n.a | n.a |
|  |  |  | Malie | 126,949 | 121,949 | 119,510 |  | 2023/08/10 | 2023/08/20 | 119,510 | 100.0 | n.a | n.a | n.a | n.a | n.a |
|  | **Total** | | | **1,305,051** | **1,342,626** | **1,315,761** | **SD** | **2023/08/10** | **2023/08/20** | **1,315,683** | **100.0** | **n.a** | **n.a** | **n.a** | **n.a** | **n.a** |
| **Total-A8** | | | | **2,241,181** | **2,275,705** | **2,230,178** | **SD** | **2023/08/10** | **2023/08/20** | **2,229,941** | **100.0** | **n.a** | **n.a** | **n.a** | **n.a** | **n.a** |
| **A9** | **Amhara** | | | | | | | | | | | | | | | |
|  |  | **Bahir Dar City** | | | | | | | | | | | | | | |
|  |  |  | Bahir Dar Town | 420,369 | 377,739 | 368,727 | SD | 2023/09/16 | 2023/09/23 | 368,649 | 100.0 | n.a | n.a | n.a | n.a | n.a |
|  |  | **Central Gonder** | | | | | | | | | | | | | | |
|  |  |  | East Dembia | 221,700 | 176,162 | 172,995 | SD | 2023/09/16 | 2023/09/23 | 172,533 | 99.7 | n.a | n.a | n.a | n.a | n.a |
|  |  | **Debre Birhan Town** | | | | | | | | | | | | | | |
|  |  |  | Debre Berhan Town | 130,940 | 154,962 | 151,439 | SD | 2023/09/16 | 2023/09/23 | 151,406 | 100.0 | n.a | n.a | n.a | n.a | n.a |
|  |  | **Gondar City** | | | | | | | | | | | | | | |
|  |  |  | Gondar Town | 470,028 | 387,385 | 376,151 | SD | 2023/09/16 | 2023/09/23 | 368,270 | 97.9 | n.a | n.a | n.a | n.a | n.a |
|  |  | **North Gojam** | | | | | | | | | | | | | | |
|  |  |  | Bahirdar Zuria | 236,667 | 198,738 | 193,945 | SD | 2023/09/16 | 2023/09/23 | 193,564 | 99.8 | n.a | n.a | n.a | n.a | n.a |
|  |  | **North Shewa** | | | | | | | | | | | | | | |
|  |  |  | Mojan Wedera | 88,634 | 76,604 | 74,188 | SD | 2023/09/16 | 2023/09/23 | 74,100 | 99.9 | n.a | n.a | n.a | n.a | n.a |
|  |  | **South Gonder** | | | | | | | | | | | | | | |
|  |  |  | Dera (AM) | 314,069 | 269,858 | 262,033 | SD | 2023/09/16 | 2023/09/23 | 261,789 | 99.9 | n.a | n.a | n.a | n.a | n.a |
|  |  | **West Gondar** | | | | | | | | | | | | | | |
|  |  |  | Metema | 174,858 | 143,267 | 139,112 | SD | 2023/09/16 | 2023/09/23 | 139,028 | 99.9 | n.a | n.a | n.a | n.a | n.a |
|  |  |  | Quara | 163,018 | 133,184 | 129,322 | SD | 2023/09/16 | 2023/09/23 | 129,133 | 99.9 | n.a | n.a | n.a | n.a | n.a |
| **Total-A9** | | | | **2,220,283** | **1,917,899** | **1,867,912** | **SD** | **2023/09/16** | **2023/09/23** | **1,858,472** | **99.5** | **n.a** | **n.a** | **n.a** | **n.a** | **n.a** |
| **A10** | **Afar** | | | | | | | | | | | | | | | |
|  |  | **Zone 3** | | | | | | | | | | | | | | |
|  |  |  | Amibara | 83,212 | 96,297 | 93,504 | SD | 2023/11/11 | 2023/11/21 | 93,418 | 99.9 | n.a | n.a | n.a | n.a | n.a |
|  |  |  | Awash | 19,189 | 150,730 | 146,359 |  | 2023/11/11 | 2023/11/21 | 146,328 | 100.0 | n.a | n.a | n.a | n.a | n.a |
|  |  |  | Awash Town | 19,409 | 71,961 | 69,874 |  | 2023/11/11 | 2023/11/21 | 69,774 | 99.9 | n.a | n.a | n.a | n.a | n.a |
|  |  |  | Dulecha | 27,175 | 95,977 | 93,194 |  | 2023/11/11 | 2023/11/21 | 93,026 | 99.8 | n.a | n.a | n.a | n.a | n.a |
|  |  |  | Hanruka | 49,556 | 42,161 | 40,938 |  | 2023/11/11 | 2023/11/21 | 40,697 | 99.4 | n.a | n.a | n.a | n.a | n.a |
|  |  | **Zone 4** | | | | | | | | | | | | | | |
|  |  |  | Teru | 62,225 | 83,475 | 81,054 | SD | 2023/11/11 | 2023/11/21 | 80,930 | 99.8 | n.a | n.a | n.a | n.a | n.a |
|  | **Total** | | | **260,766** | **540,601** | **524,923** | **SD** | **2023/11/11** | **2023/11/21** | **524,173** | **99.9** | **n.a** | **n.a** | **n.a** | **n.a** | **n.a** |
|  | **Sidama** | | | | | | | | | | | | | | | |
|  |  | **Sidama** | | | | | | | | | | | | | | |
|  |  |  | Dara Otilicho | 118,814 | 114,134 | 110,824 | SD | 2023/11/11 | 2023/11/21 | 110,705 | 99.9 | n.a | n.a | n.a | n.a | n.a |
|  |  |  | Wondo-Genet | 167,115 | 203,675 | 197,768 |  | 2023/11/11 | 2023/11/21 | 197,709 | 100.0 | n.a | n.a | n.a | n.a | n.a |
|  |  |  | Wondo-Genet Town | 22,562 | 58,595 | 56,896 |  | 2023/11/11 | 2023/11/21 | 56,856 | 99.9 | n.a | n.a | n.a | n.a | n.a |
|  | **Total** | | | **308,491** | **376,404** | **365,488** | **SD** | **2023/11/11** | **2023/11/21** | **365,270** | **99.9** | **n.a** | **n.a** | **n.a** | **n.a** | **n.a** |
|  | **SNNPR** | | | | | | | | | | | | | | | |
|  |  | **Gamo** | | | | | | | | | | | | | | |
|  |  |  | Arba Minch Zuria | 137,296 | 131,889 | 128,064 | SD | 2023/11/11 | 2023/11/21 | 127,899 | 99.9 | n.a | n.a | n.a | n.a | n.a |
|  |  | **Gedeo** | | | | | | | | | | | | | | |
|  |  |  | Chelelektu Town | 30,702 | 59,493 | 57,768 | SD | 2023/11/11 | 2023/11/21 | 57,609 | 99.7 | n.a | n.a | n.a | n.a | n.a |
|  |  |  | Dila Town | 147,289 | 99,188 | 96,312 |  | 2023/11/11 | 2023/11/21 | 96,183 | 99.9 | n.a | n.a | n.a | n.a | n.a |
|  |  | **Gofa** | | | | | | | | | | | | | | |
|  |  |  | Zala | 111,809 | 107,403 | 104,288 | SD | 2023/11/11 | 2023/11/21 | 104,199 | 99.9 | n.a | n.a | n.a | n.a | n.a |
|  |  | **Gurage** | | | | | | | | | | | | | | |
|  |  |  | Sodo | 142,560 | 254,604 | 247,220 | SD | 2023/11/11 | 2023/11/21 | 247,074 | 99.9 | n.a | n.a | n.a | n.a | n.a |
|  | **Total** | | | **569,656** | **652,577** | **633,652** | **SD** | **2023/11/11** | **2023/11/21** | **632,964** | **99.9** | **n.a** | **n.a** | **n.a** | **n.a** | **n.a** |
| **Total-A10** | | | | **1,138,913** | **1,569,582** | **1,524,063** | **SD** | **2023/11/11** | **2023/11/21** | **1,522,407** | **99.9** | **n.a** | **n.a** | **n.a** | **n.a** | **n.a** |
| **A11** | **Amhara** | | | | | | | | | | | | | | | |
|  |  | **North Wollo** | | | | | | | | | | | | | | |
|  |  |  | Gazgibla | 87,052 | 57,365 | 40,551 | SD | 2023/11/29 | 2023/12/05 | 40,386 | 99.6 | n.a | n.a | n.a | n.a | n.a |
|  |  | **Waghimra** | | | | | | | | | | | | | | |
|  |  |  | Kobo Town | 57,473 | 155,114 | 90,583 | SD | 2023/11/29 | 2023/12/05 | 90,417 | 99.8 | n.a | n.a | n.a | n.a | n.a |
|  | **Total** | | | **144,525** | **212,479** | **131,134** | **SD** | **2023/11/29** | **2023/12/05** | **130,803** | **99.7** | **n.a** | **n.a** | **n.a** | **n.a** | **n.a** |
|  | **Oromia** | | | | | | | | | | | | | | | |
|  |  | **East Hararge** | | | | | | | | | | | | | | |
|  |  |  | Bedeno | 358,872 | 60,901 | 56,178 | SD | 2023/11/29 | 2023/12/05 | 56,172 | 100.0 | n.a | n.a | n.a | n.a | n.a |
|  |  |  | Girawa | 359,887 | 65,591 | 60,504 | SD | 2023/11/29 | 2023/12/05 | 60,504 | 100.0 | n.a | n.a | n.a | n.a | n.a |
|  |  |  | Gole Oda | 123,504 | 56,418 | 52,043 | SD | 2023/11/29 | 2023/12/05 | 52,043 | 100.0 | n.a | n.a | n.a | n.a | n.a |
|  |  |  | Haromaya | 320,687 | 88,312 | 81,463 | SD | 2023/11/29 | 2023/12/05 | 81,463 | 100.0 | n.a | n.a | n.a | n.a | n.a |
|  |  |  | Kumbi | 55,721 | 55,721 | 51,552 | SD | 2023/11/29 | 2023/12/05 | 51,552 | 100.0 | n.a | n.a | n.a | n.a | n.a |
|  |  |  | Kurfa Chele | 89,015 | 92,670 | 84,860 | SD | 2023/11/29 | 2023/12/05 | 84,860 | 100.0 | n.a | n.a | n.a | n.a | n.a |
|  |  |  | Kersa | 257,714 | 95,344 | 87,950 | SD | 2023/11/29 | 2023/12/05 | 87,948 | 100.0 | n.a | n.a | n.a | n.a | n.a |
|  |  |  | Mayu Muluke | 69,744 | 72,422 | 66,806 | SD | 2023/11/29 | 2023/12/05 | 66,806 | 100.0 | n.a | n.a | n.a | n.a | n.a |
|  |  | **West Guji** | | | | | | | | | | | | | | |
|  |  |  | Abaya | 160,149 | 108,000 | 99,785 | SD | 2023/11/29 | 2023/12/05 | 99,785 | 100.0 | n.a | n.a | n.a | n.a | n.a |
|  |  |  | Gelana | 108,514 | 98,000 | 90,400 | SD | 2023/11/29 | 2023/12/05 | 90,390 | 100.0 | n.a | n.a | n.a | n.a | n.a |
|  | **Total** | | | **1,903,807** | **793,379** | **731,541** | **SD** | **2023/11/29** | **2023/12/05** | **731,523** | **100.0** | **n.a** | **n.a** | **n.a** | **n.a** | **n.a** |
| **Total-A11** | | | | **2,048,332** | **1,005,858** | **862,675** | **SD** | **2023/11/29** | **2023/12/05** | **862,326** | **100.0** | **n.a** | **n.a** | **n.a** | **n.a** | **n.a** |
| **Total-A1 to A11 (including vaccination using retrieved vaccines)** | | | | **22,273,582** | **15,965,523** | **15,229,535** | **n.a** | **n.a** | **n.a** | **13,911,605** | **91.3** | **n.a** | **n.a** | **n.a** | **4,002,915** | **96.8** |
| **Grand Total** | | | | **23,476,956** | **16,291,281** | **15,540,271** | **n.a** | **n.a** | **n.a** | **14,215,949** | **91.5** | **n.a** | **n.a** | **n.a** | **4,103,848** | **96.9** |

**Footnote:**

^1^ Request number refers to each OCV request made by the Ethiopian government.

^2^ Zone is the second administrative division in Ethiopia, subdivided from Region.

^3^ Woreda is the third administrative division in Ethiopia, subdivided from Zone.

^4^ Total population in the corresponding year when vaccination was conducted.

^5^ Population of OCV vaccination targeted kebeles in each Woreda in the corresponding year when vaccination was conducted. Some campaigns targeted both entire population and IDP in the woredas generating larger number of targeted population than total population in the woredas.

^6^ In first round. OCV target population is the population of OCV vaccination targeted kebeles in each woreda excluding infants (i.e., < 1 year). Some campaigns targeted both entire population and IDP in the woredas generating larger number of targeted population than total population in the woredas.

^7^ OCV coverage (%) was calculated based on the actual number of people vaccinated in each round (numerator) out of the OCV target population (denominator) in each round.

^8^ Bilateral: Ethiopian government’s bilateral request to the government of the Republic of Korea for OCV doses for cholera outbreak control in 2019.

^9^ SNNPR: Southern Nations, Nationalities, and Peoples’ Region^1010^ n.a: not applicable.

^11^ ECCP: ‘Ethiopia Cholera Control and Prevention’ Project. Post-campaign coverage survey was conducted: coverage of full 2 doses was 78% in Shashemene Town and 83.06% in Shashemene Woreda % (**REF: Se Eun Park. Pre-emptive oral cholera vaccine (OCV) mass vaccination campaign in cholera high priority hotspots in Shashemene Town and Woreda, West Arsi Zone, Oromia Region, Ethiopia; included in this CID supplement**).

^12^ One of the homeless shelters in Addis Ababa.

^13^ IPDC: Industrial Park Development Corporation.

^14^ Total population in 2020 when 1^st^ round was conducted.

^15^ Total population represents the number of people in the community. Targeted population of preventive vaccination campaign conducted in Tigray in 2021 was the community and internally displaced people (IDP). Therefore, Population of targeted kebeles and OCV target population reflect the combined number of the community and IDP targeted by preventive vaccination campaigns.

^16^ Total population in 2021 when 1^st^ round was conducted.

^17^ It used remaining OCV doses from previous OCV vaccination campaigns.

^18^ IDP: Internally Displaced People

^19^ Target population included community and IDP.

**Supplementary** **Table 2. Detailed age group and sex stratified data on OCV vaccination campaigns in Ethiopia from 2019 to 2023**

| **Request Number^1^** | **Region/Zone^2^/Woreda(District)^3^** | | | **1^st^ Round** | | | | | | | | | **2^nd^ Round** | | | | | | | | |
| --- | --- | --- | --- | --- | --- | --- | --- | --- | --- | --- | --- | --- | --- | --- | --- | --- | --- | --- | --- | --- | --- |
|  |  |  |  | **Campaign Start date**  **(YYYY/MM/DD)** | **Campaign End date (YYYY/MM/DD)** | **Number of people vaccinated with OCV (n)^4^** | **1-4 years^5^** | | **5-14 years^6^** | | ≥ **15 years** | | **Campaign Start date (YYYY/MM/DD)** | **Campaign End date (YYYY/MM/DD)** | **Number ofpeople vaccinated with OCV(n)** | **1-4 years** | | **5-14 years** | | ≥ **15 years** | |
|  |  |  |  |  |  |  | **Male** | **Female** | **Male** | **Female** | **Male** | **Female** |  |  |  | **Male** | **Female** | **Male** | **Female** | **Male** | **Female** |
| **Bilateral^7^** | **SNNPR^8^** | | | | | | | | | | | | | | | | | | | | |
|  |  | **Gamo** | | | | | | | | | | | | | | | | | | | |
|  |  |  | Kamba | 2019/07/01 | 2019/07/05 | 12,148 | n.a^9^ | n.a | n.a | n.a | n.a | n.a | n.a | n.a | n.a | n.a | n.a | n.a | n.a | n.a | n.a |
|  |  | **Goda** | | | | | | | | | | | | | | | | | | | |
|  |  |  | Uba Debretsehay | 2019/07/01 | 2019/07/05 | 17,820 | n.a | n.a | n.a | n.a | n.a | n.a | n.a | n.a | n.a | n.a | n.a | n.a | n.a | n.a | n.a |
|  |  | **Gofa** | | | | | | | | | | | | | | | | | | | |
|  |  |  | Zala | 2019/07/01 | 2019/07/05 | 14,065 | n.a | n.a | n.a | n.a | n.a | n.a | n.a | n.a | n.a | n.a | n.a | n.a | n.a | n.a | n.a |
|  |  | **South Omo** | | | | | | | | | | | | | | | | | | | |
|  |  |  | Selemago | 2019/07/01 | 2019/07/05 | 26,196 | n.a | n.a | n.a | n.a | n.a | n.a | n.a | n.a | n.a | n.a | n.a | n.a | n.a | n.a | n.a |
|  | **Total** | | | **2019/07/01** | **2019/07/05** | **70,229** | **n.a** | **n.a** | **n.a** | **n.a** | **n.a** | **n.a** | **n.a** | **n.a** | **n.a** | **n.a** | **n.a** | **n.a** | **n.a** | **n.a** | **n.a** |
|  | **Somali** | | | | | | | | | | | | | | | | | | | | |
|  |  | **Shebele** | | | | | | | | | | | | | | | | | | | |
|  |  |  | Hudet | 2019/07/01 | 2019/07/05 | 32,286 | n.a | n.a | n.a | n.a | n.a | n.a | n.a | n.a | n.a | n.a | n.a | n.a | n.a | n.a | n.a |
|  |  |  | Kelafo | 2019/07/01 | 2019/07/05 | 54,231 | n.a | n.a | n.a | n.a | n.a | n.a | n.a | n.a | n.a | n.a | n.a | n.a | n.a | n.a | n.a |
|  |  | **Siti** | | | | | | | | | | | | | | | | | | | |
|  |  |  | Erer | 2019/07/01 | 2019/07/05 | 46,040 | n.a | n.a | n.a | n.a | n.a | n.a | n.a | n.a | n.a | n.a | n.a | n.a | n.a | n.a | n.a |
|  | **Total** | | | **2019/07/01** | **2019/07/05** | **132,557** | **n.a** | **n.a** | **n.a** | **n.a** | **n.a** | **n.a** | **n.a** | **n.a** | **n.a** | **n.a** | **n.a** | **n.a** | **n.a** | **n.a** | **n.a** |
| **Total-Bilateral** | | | | **2019/07/01** | **2019/07/05** | **202,786** | **n.a** | **n.a** | **n.a** | **n.a** | **n.a** | **n.a** | **n.a** | **n.a** | **n.a** | **n.a** | **n.a** | **n.a** | **n.a** | **n.a** | **n.a** |
| **ECCP^10^** | **Oromia** | | | | | | | | | | | | | | | | | | | | |
|  |  | **West Arsi** | | | | | | | | | | | | | | | | | | | |
|  |  |  | Shashemene Town | 2022/05/11 | 2022/05/16 | 41,056 | 4,526 | 4,804 | 6,363 | 7,219 | 7,770 | 10,374 | 2022/05/27 | 2022/05/30 | 40,453 | 4,417 | 5,255 | 6,816 | 7,181 | 7,387 | 9,397 |
|  |  |  | Shashemene Woreda | 2022/05/11 | 2022/05/16 | 60,502 | 6,944 | 7,123 | 12,184 | 11,641 | 10,188 | 12,422 | 2022/05/27 | 2022/06/01 | 60,480 | 7,237 | 7,562 | 11,410 | 11,608 | 10,702 | 11,961 |
| **Total-ECCP** | | | | **2022/05/11** | **2022/05/16** | **101,558** | **11,470** | **11,927** | **18,547** | **18,860** | **17,958** | **22,796** | **2022/05/27** | **2022/06/01** | **100,933** | **11,654** | **12,817** | **18,226** | **18,789** | **18,089** | **21,358** |
| **Total-outside of global stockpile** | | | | **n.a** | **n.a** | **304,344** | **11,470** | **11,927** | **18,547** | **18,860** | **17,958** | **22,796** | **n.a** | **n.a** | **100,933** | **11,654** | **12,817** | **18,226** | **18,789** | **18,089** | **21,358** |
| **A1** | **Oromia** | | | | | | | | | | | | | | | | | | | | |
|  |  | **West Hararge** | | | | | | | | | | | | | | | | | | | |
|  |  |  | Bedessa | 2019/07/01 | 2019/07/05 | 27,136 | 3,529 | 4,120 | 7,316 | 8,336 | 1,922 | 1,913 | 2019/12/14 | 2019/12/24 | 28,881 | 4,059 | 4,047 | 5,693 | 6,304 | 4,458 | 4,320 |
|  |  |  | Chiro | 2019/07/01 | 2019/07/05 | 98,532 | 8,822 | 9,177 | 16,931 | 17,905 | 22,366 | 23,331 | 2019/12/14 | 2019/12/24 | 96,281 | 9,507 | 10,103 | 17,687 | 19,727 | 18,376 | 20,881 |
|  |  |  | Gemechise | 2019/07/01 | 2019/07/05 | 28,998 | 2,545 | 2,602 | 10,007 | 10,507 | 1,753 | 1,584 | 2019/12/14 | 2019/12/24 | 55,762 | 3,702 | 3,640 | 8,061 | 7,453 | 15,753 | 17,153 |
|  |  |  | Mieso | 2019/07/01 | 2019/07/05 | 78,784 | 10,006 | 8,645 | 14,762 | 13,256 | 17,021 | 15,094 | 2019/12/14 | 2019/12/24 | 79,337 | 9,215 | 8,947 | 18,487 | 18,208 | 12,957 | 11,523 |
|  |  |  | Oda Bultum | 2019/07/01 | 2019/07/05 | 57,290 | 4,057 | 4,154 | 8,594 | 7,786 | 15,845 | 16,854 | 2019/12/14 | 2019/12/24 | 25,942 | 3,472 | 3,275 | 7,035 | 7,955 | 2,106 | 2,099 |
|  | **Total** | | | **2019/07/01** | **2019/07/05** | **290,740** | **28,959** | **28,698** | **57,610** | **57,790** | **58,907** | **58,776** | **2019/12/14** | **2019/12/24** | **286,203** | **29,955** | **30,012** | **56,963** | **59,647** | **53,650** | **55,976** |
|  | **Addis Ababa** | | | | | | | | | | | | | | | | | | | | |
|  |  | **Camps^11^** | | | | | | | | | | | | | | | | | | | |
|  |  |  | Camps | 2019/07/01 | 2019/07/05 | 17,324 | 405 | 320 | 4,208 | 3,770 | 4,500 | 4,121 | n.a | n.a | n.a | n.a | n.a | n.a | n.a | n.a | n.a |
|  | **Total** | | | **2019/07/01** | **2019/07/05** | **17,324** | **405** | **320** | **4,208** | **3,770** | **4,500** | **4,121** | **n.a** | **n.a** | **n.a** | **n.a** | **n.a** | **n.a** | **n.a** | **n.a** | **n.a** |
|  | **Afar** | | | | | | | | | | | | | | | | | | | | |
|  |  | **Zone 3** | | | | | | | | | | | | | | | | | | | |
|  |  |  | Amibara | 2019/07/01 | 2019/07/05 | 51,933 | 3,283 | 3,283 | 9,530 | 9,530 | 16,488 | 9,819 | n.a | n.a | n.a | n.a | n.a | n.a | n.a | n.a | n.a |
|  |  | **Zone 4** | | | | | | | | | | | | | | | | | | | |
|  |  |  | Ewa | 2019/07/01 | 2019/07/05 | 29,448 | 2,715 | 2,715 | 8,331 | 8,331 | 3,796 | 3,560 | n.a | n.a | n.a | n.a | n.a | n.a | n.a | n.a | n.a |
|  | **Total** | | | **2019/07/01** | **2019/07/05** | **81,381** | **5,998** | **5,998** | **17,861** | **17,861** | **20,284** | **13,379** | **n.a** | **n.a** | **n.a** | **n.a** | **n.a** | **n.a** | **n.a** | **n.a** | **n.a** |
|  | **Sidama** | | | | | | | | | | | | | | | | | | | | |
|  |  | **Hawassa** | | | | | | | | | | | | | | | | |  |  |  |
|  |  |  | Hawassa/IPDC^12^ | 2019/07/01 | 2019/07/05 | 23,322 | 0 | 0 | 810 | 800 | 10,212 | 11,500 | n.a | n.a | n.a | n.a | n.a | n.a | n.a | n.a | n.a |
|  | **Total** | | | **2019/07/01** | **2019/07/05** | **23,322** | **0** | **0** | **810** | **800** | **10,212** | **11,500** | **n.a** | **n.a** | **n.a** | **n.a** | **n.a** | **n.a** | **n.a** | **n.a** | **n.a** |
|  | **Total-A1** | | | **2019/07/01** | **2019/07/05** | **412,767** | **35,362** | **35,016** | **80,489** | **80,221** | **93,903** | **87,776** | **2019/12/14** | **2019/12/24** | **286,203** | **29,955** | **30,012** | **56,963** | **59,647** | **53,650** | **55,976** |
| **A2** | **Gambella** | | | | | | | | | | | | | | | | | | | | |
|  |  | **Agnuak** | | | | | | | | | | | | | | | | | | | |
|  |  |  | Dima | 2020/12/21 | 2020/12/29 | 24,226 | 1,487 | 1,546 | 3,111 | 3,107 | 8,868 | 6,107 | 2021/02/28 | 2021/03/06 | 24,226 | 1,487 | 1,546 | 3,111 | 3,107 | 8,868 | 6,107 |
|  | **Total** | | | **2020/12/21** | **2020/12/29** | **24,226** | **1,487** | **1,546** | **3,111** | **3,107** | **8,868** | **6,107** | **2021/02/28** | **2021/03/06** | **24,226** | **1,487** | **1,546** | **3,111** | **3,107** | **8,868** | **6,107** |
|  | **Oromia** | | | | | | | | | | | | | | | | | | | | |
|  |  | **Guji** | | | | | | | | | | | | | | | | | | | |
|  |  |  | Adola Rede | 2020/12/21 | 2020/12/29 | 143,477 | 13,607 | 13,855 | 25,119 | 24,585 | 34,895 | 31,416 | 2021/02/28 | 2021/03/06 | 137,876 | 12,378 | 13,855 | 23,568 | 20,786 | 35,873 | 31,416 |
|  |  |  | Liben | 2020/12/21 | 2020/12/29 | 35,703 | 3,501 | 3,676 | 6,891 | 6,977 | 7,158 | 7,500 | 2021/02/28 | 2021/03/06 | 35,703 | 3,501 | 3,676 | 6,891 | 6,977 | 7,158 | 7,500 |
|  |  |  | Negele Town | 2020/12/21 | 2020/12/29 | 54,075 | 3,865 | 3,335 | 7,193 | 7,440 | 17,442 | 14,800 | 2021/02/28 | 2021/03/06 | 54,075 | 3,865 | 3,335 | 7,193 | 7,440 | 17,442 | 14,800 |
|  |  | **West Arsi** | | | | | | | | | | | | | | | | | | | |
|  |  |  | Nensebo | 2020/12/21 | 2020/12/29 | 32,745 | 2,942 | 3,125 | 5,379 | 5,928 | 7,108 | 8,263 | 2021/02/28 | 2021/03/06 | 32,745 | 2,942 | 3,125 | 5,379 | 5,928 | 7,108 | 8,263 |
|  |  | **West Guji** | | | | | | | | | | | | | | | | | | | |
|  |  |  | Abaya | 2020/12/21 | 2020/12/29 | 145,488 | 13,068 | 13,114 | 25,078 | 25,685 | 33,316 | 35,227 | 2021/02/28 | 2021/03/06 | 149,849 | 14,520 | 13,114 | 27,987 | 25,685 | 33,316 | 35,227 |
|  |  |  | Gelana | 2020/12/21 | 2020/12/29 | 59,690 | 4,764 | 4,922 | 6,625 | 7,288 | 13,222 | 22,869 | 2021/02/28 | 2021/03/06 | 59,690 | 4,764 | 4,922 | 6,625 | 7,288 | 13,222 | 22,869 |
|  |  |  | Melkasoda | 2020/12/21 | 2020/12/29 | 12,403 | 1,152 | 1,006 | 2,311 | 1,532 | 3,705 | 2,697 | 2021/02/28 | 2021/03/06 | 12,403 | 1,152 | 1,006 | 2,311 | 1,532 | 3,705 | 2,697 |
|  | **Total** | | | **2020/12/21** | **2020/12/29** | **483,581** | **42,899** | **43,033** | **78,596** | **79,435** | **116,846** | **122,772** | **2021/02/28** | **2021/03/06** | **482,341** | **43,122** | **43,033** | **79,954** | **75,636** | **117,824** | **122,772** |
|  | **Sidama** | | | | | | | | | | | | | | | | | | | | |
|  |  | **Sidama** | | | | | | | | | | | | | | | | | | | |
|  |  |  | Aroresa | 2020/12/21 | 2020/12/29 | 89,696 | 6,495 | 6,664 | 15,682 | 15,787 | 22,662 | 22,406 | 2021/02/28 | 2021/03/06 | 92,289 | 7,035 | 5,798 | 17,230 | 15,787 | 23,456 | 22,983 |
|  |  |  | Bona Zuria | 2020/12/21 | 2020/12/29 | 135,319 | 3,761 | 4,334 | 17,809 | 18,536 | 45,038 | 45,841 | 2021/02/28 | 2021/03/06 | 133,995 | 2,437 | 4,334 | 17,809 | 18,536 | 45,038 | 45,841 |
|  |  |  | Chabe Gambiltu | 2020/12/21 | 2020/12/29 | 45,178 | 2,331 | 2,384 | 9,739 | 10,024 | 10,062 | 10,638 | 2021/02/28 | 2021/03/06 | 43,725 | 3,412 | 1,598 | 9,723 | 10,784 | 8,700 | 9,508 |
|  |  |  | Huko | 2020/12/21 | 2020/12/29 | 81,996 | 6,281 | 6,551 | 20,013 | 19,221 | 15,277 | 14,653 | 2021/02/28 | 2021/03/06 | 81,836 | 6,206 | 5,201 | 19,650 | 20,167 | 14,823 | 15,789 |
|  | **Total** | | | **2020/12/21** | **2020/12/29** | **352,189** | **18,868** | **19,933** | **63,243** | **63,568** | **93,039** | **93,538** | **2021/02/28** | **2021/03/06** | **351,845** | **19,090** | **16,931** | **64,412** | **65,274** | **92,017** | **94,121** |
|  | **SNNPR** | | | | | | | | | | | | | | | | | | | | |
|  |  | **Dawuro** | | | | | | | | | | | | | | | | | | | |
|  |  |  | Gena | 2020/12/21 | 2020/12/29 | 39,982 | 2,582 | 2,935 | 6,834 | 7,255 | 9,619 | 10,757 | 2021/02/28 | 2021/03/06 | 39,982 | 2,582 | 2,935 | 6,834 | 7,255 | 9,619 | 10,757 |
|  |  |  | Tercha Town | 2020/12/21 | 2020/12/29 | 22,880 | 1,087 | 1,140 | 2,920 | 3,439 | 7,631 | 6,663 | 2021/02/28 | 2021/03/06 | 22,880 | 1,087 | 1,140 | 2,920 | 3,439 | 7,631 | 6,663 |
|  |  |  | Tercha Zuria | 2020/12/21 | 2020/12/29 | 54,560 | 2,416 | 2,677 | 8,700 | 8,417 | 16,017 | 16,333 | 2021/02/28 | 2021/03/06 | 53,099 | 2,416 | 2,677 | 7,239 | 8,417 | 16,017 | 16,333 |
|  |  | **South Omo** | | | | | | | | | | | | | | | | | | | |
|  |  |  | Benatsemay | 2020/12/21 | 2020/12/29 | 29,752 | 4,490 | 2,615 | 4,585 | 4,647 | 6,514 | 6,901 | 2021/02/28 | 2021/03/06 | 24,207 | 2,609 | 1,283 | 4,209 | 3,903 | 6,112 | 6,091 |
|  |  |  | Dasenech | 2020/12/21 | 2020/12/29 | 69,675 | 4,769 | 4,630 | 11,733 | 11,381 | 18,394 | 18,768 | 2021/02/28 | 2021/03/06 | 66,974 | 4,769 | 4,630 | 11,733 | 10,782 | 16,967 | 18,093 |
|  |  |  | Hamer | 2020/12/21 | 2020/12/29 | 38,034 | 3,017 | 3,007 | 6,449 | 5,962 | 9,886 | 9,713 | 2021/02/28 | 2021/03/06 | 33,476 | 1,703 | 2,084 | 4,128 | 5,962 | 9,886 | 9,713 |
|  |  |  | Mallei | 2020/12/21 | 2020/12/29 | 74,164 | 6,264 | 6,077 | 13,292 | 14,483 | 15,412 | 18,636 | 2021/02/28 | 2021/03/06 | 66,532 | 4,187 | 5,081 | 11,782 | 12,901 | 15,283 | 17,298 |
|  |  |  | Ngangatom | 2020/12/21 | 2020/12/29 | 10,929 | 976 | 976 | 1,526 | 1,550 | 2,968 | 2,933 | 2021/02/28 | 2021/03/06 | 10,929 | 976 | 976 | 1,526 | 1,550 | 2,968 | 2,933 |
|  |  | **West Omo** | | | | | | | | | | | | | | | | | | | |
|  |  |  | Bero | 2020/12/21 | 2020/12/29 | 19,706 | 1,668 | 1,715 | 2,911 | 2,878 | 5,128 | 5,406 | 2021/02/28 | 2021/03/06 | 19,318 | 1,668 | 1,327 | 2,911 | 2,878 | 5,128 | 5,406 |
|  |  |  | Gachit | 2020/12/21 | 2020/12/29 | 57,308 | 5,889 | 5,785 | 8,816 | 8,966 | 13,973 | 13,879 | 2021/02/28 | 2021/03/06 | 55,077 | 5,889 | 5,785 | 8,816 | 6,735 | 13,973 | 13,879 |
|  |  |  | Menit Goldiya | 2020/12/21 | 2020/12/29 | 75,822 | 6,487 | 6,521 | 5,300 | 5,577 | 25,693 | 26,244 | 2021/02/28 | 2021/03/06 | 75,822 | 6,487 | 6,521 | 5,300 | 5,577 | 25,693 | 26,244 |
|  |  |  | Menit Shasha | 2020/12/21 | 2020/12/29 | 35,877 | 2,848 | 2,807 | 6,114 | 6,732 | 8,661 | 8,715 | 2021/02/28 | 2021/03/06 | 35,877 | 2,848 | 2,807 | 6,114 | 6,732 | 8,661 | 8,715 |
|  |  |  | Surma | 2020/12/21 | 2020/12/29 | 30,916 | 2,057 | 2,529 | 4,593 | 5,027 | 8,052 | 8,658 | 2021/02/28 | 2021/03/06 | 30,026 | 1,167 | 2,529 | 4,593 | 5,027 | 8,052 | 8,658 |
|  | **Total** | | | **2020/12/21** | **2020/12/29** | **559,605** | **44,550** | **43,414** | **83,773** | **86,314** | **147,948** | **153,606** | **2021/02/28** | **2021/03/06** | **534,199** | **38,388** | **39,775** | **78,105** | **81,158** | **145,990** | **150,783** |
|  | **Somali** | | | | | | | | | | | | | | | | | | | | |
|  |  | **Shebele** | | | | | | | | | | | | | | | | | | | |
|  |  |  | Fer Fer | 2020/12/21 | 2020/12/29 | 24,621 | 2,020 | 3,105 | 6,412 | 7,419 | 1,715 | 3,950 | 2021/02/28 | 2021/03/06 | 24,677 | 2,045 | 2,484 | 5,291 | 5,618 | 4,405 | 4,834 |
|  |  |  | Kelafo | 2020/12/21 | 2020/12/29 | 45,686 | 4,560 | 4,943 | 7,027 | 7,223 | 10,374 | 11,559 | 2021/02/28 | 2021/03/06 | 45,506 | 4,560 | 4,943 | 7,027 | 7,223 | 10,284 | 11,469 |
|  |  |  | Mustahkin | 2020/12/21 | 2020/12/29 | 54,556 | 2,209 | 2,211 | 10,503 | 13,524 | 14,487 | 11,622 | 2021/02/28 | 2021/03/06 | 52,420 | 3,585 | 2,815 | 8,339 | 11,794 | 11,823 | 14,064 |
|  | **Total** | | | **2020/12/21** | **2020/12/29** | **124,863** | **8,789** | **10,259** | **23,942** | **28,166** | **26,576** | **27,131** | **2021/02/28** | **2021/03/06** | **122,603** | **10,190** | **10,242** | **20,657** | **24,635** | **26,512** | **30,367** |
| **Total-A2** | | | | **2020/12/21** | **2020/12/29** | **1,544,464** | **116,593** | **118,185** | **252,665** | **260,590** | **393,277** | **403,154** | **2021/02/28** | **2021/03/06** | **1,515,214** | **112,277** | **111,527** | **246,239** | **249,810** | **391,211** | **404,150** |
| **A3** | **Oromia** | | | | | | | | | | | | | | | | | | | | |
|  |  | **West Arsi** | | | | | | | | | | | | | | | | | | | |
|  |  |  | Nensebo | 2021/05/20 | 2021/05/26 | 148,201 | 8,300 | 8,639 | 24,364 | 25,358 | 40,354 | 41,186 | 2021/08/18 | 2021/08/25 | 148,201 | 8,300 | 8,639 | 24,364 | 25,358 | 40,354 | 41,186 |
|  | **Total** | | | **2021/05/20** | **2021/05/26** | **148,201** | **8,300** | **8,639** | **24,364** | **25,358** | **40,354** | **41,186** | **2021/08/18** | **2021/08/25** | **148,201** | **8,300** | **8,639** | **24,364** | **25,358** | **40,354** | **41,186** |
|  | **SNNPR** | | | | | | | | | | | | | | | | | | | | |
|  |  | **Bench Sheko Zone** | | | | | | | | | | | | | | | | | | | |
|  |  |  | Mizan Aman | 2021/05/20 | 2021/05/26 | 71,758 | 4,019 | 4,183 | 11,797 | 12,278 | 19,539 | 19,942 | 2021/08/18 | 2021/08/25 | 71,758 | 4,019 | 4,183 | 11,797 | 12,278 | 19,539 | 19,942 |
|  |  |  | Semen Bench | 2021/05/20 | 2021/05/26 | 92,696 | 5,940 | 6,151 | 15,987 | 16,609 | 25,988 | 22,021 | 2021/08/18 | 2021/08/25 | 92,696 | 5,192 | 5,403 | 15,239 | 15,861 | 25,240 | 25,761 |
|  |  |  | Shebench | 2021/05/20 | 2021/05/26 | 145,818 | 8,167 | 8,500 | 23,972 | 24,950 | 39,705 | 40,524 | 2021/08/18 | 2021/08/25 | 145,118 | 8,128 | 8,459 | 23,857 | 24,830 | 39,515 | 40,329 |
|  |  |  | Sheko | 2021/05/20 | 2021/05/26 | 65,507 | 3,669 | 3,819 | 10,769 | 11,208 | 17,477 | 18,565 | 2021/08/18 | 2021/08/25 | 64,515 | 3,613 | 3,761 | 10,606 | 11,039 | 17,567 | 17,929 |
|  |  |  | Siz Town | 2021/05/20 | 2021/05/26 | 45,815 | 2,566 | 2,671 | 7,532 | 7,839 | 12,224 | 12,983 | 2021/08/18 | 2021/08/25 | 45,815 | 2,566 | 2,671 | 7,532 | 7,839 | 12,475 | 12,732 |
|  |  |  | South Bench | 2021/05/20 | 2021/05/26 | 148,044 | 8,292 | 8,630 | 24,338 | 25,331 | 39,497 | 41,956 | 2021/08/18 | 2021/08/25 | 148,044 | 8,292 | 8,630 | 24,338 | 25,331 | 40,311 | 41,142 |
|  |  | **Dawro** | | | | | | | | | | | | | | | | | | | |
|  |  |  | Gena | 2021/05/20 | 2021/05/26 | 44,120 | 2,471 | 2,572 | 7,253 | 7,549 | 12,014 | 12,261 | 2021/08/18 | 2021/08/25 | 44,019 | 2,465 | 2,566 | 7,237 | 7,532 | 11,986 | 12,233 |
|  |  |  | Isara | 2021/05/20 | 2021/05/26 | 83,356 | 4,669 | 4,859 | 13,703 | 14,263 | 22,697 | 23,165 | 2021/08/18 | 2021/08/25 | 83,314 | 4,666 | 4,857 | 13,696 | 14,255 | 22,686 | 23,154 |
|  |  |  | Kechi | 2021/05/20 | 2021/05/26 | 29,966 | 1,678 | 1,747 | 4,926 | 5,127 | 8,160 | 8,328 | 2021/08/18 | 2021/08/25 | 29,902 | 1,675 | 1,743 | 4,916 | 5,116 | 8,142 | 8,310 |
|  |  |  | Mari Mansa | 2021/05/20 | 2021/05/26 | 74,555 | 4,176 | 4,346 | 12,256 | 12,757 | 20,301 | 20,719 | 2021/08/18 | 2021/08/25 | 73,556 | 4,120 | 4,288 | 12,092 | 12,586 | 20,029 | 20,441 |
|  |  |  | Tercha Town admin | 2021/05/20 | 2021/05/26 | 24,892 | 1,394 | 1,451 | 4,092 | 4,259 | 6,778 | 6,918 | 2021/08/18 | 2021/08/25 | 24,892 | 1,394 | 1,451 | 4,092 | 4,259 | 6,778 | 6,918 |
|  |  |  | Tercha Zuriya | 2021/05/20 | 2021/05/26 | 67,641 | 3,788 | 3,943 | 11,120 | 11,574 | 18,418 | 18,798 | 2021/08/18 | 2021/08/25 | 67,641 | 3,788 | 3,943 | 11,120 | 11,574 | 18,418 | 18,798 |
|  |  |  | Tocha | 2021/05/20 | 2021/05/26 | 125,570 | 7,033 | 7,320 | 20,643 | 21,485 | 34,192 | 34,897 | 2021/08/18 | 2021/08/25 | 125,168 | 7,010 | 7,296 | 20,577 | 21,417 | 34,083 | 34,785 |
|  | **Total** | | | **2021/05/20** | **2021/05/26** | **1,019,738** | **57,862** | **60,192** | **168,388** | **175,229** | **276,990** | **281,077** | **2021/08/18** | **2021/08/25** | **1,016,438** | **56,928** | **59,251** | **167,099** | **173,917** | **276,769** | **282,474** |
| **Total-A3** | | | | **2021/05/20** | **2021/05/26** | **1,167,939** | **66,162** | **68,831** | **192,752** | **200,587** | **317,344** | **322,263** | **2021/08/18** | **2021/08/25** | **1,164,639** | **65,228** | **67,890** | **191,463** | **199,275** | **317,123** | **323,660** |
| **A4** | **Tigray** | | | | | | | | | | | | | | | | | | | | |
|  |  | **Central** | | | | | | | | | | | | | | | | | | | |
|  |  |  | AbiAdi Town | 2021/06/10 | 2021/06/17 | 25,780 | n.a | n.a | n.a | n.a | n.a | n.a | n.a | n.a | n.a | n.a | n.a | n.a | n.a | n.a | n.a |
|  |  |  | Adwa Town | 2021/06/10 | 2021/06/17 | 70,762 | n.a | n.a | n.a | n.a | n.a | n.a | n.a | n.a | n.a | n.a | n.a | n.a | n.a | n.a | n.a |
|  |  |  | Axum Town | 2021/06/10 | 2021/06/17 | 74,101 | n.a | n.a | n.a | n.a | n.a | n.a | n.a | n.a | n.a | n.a | n.a | n.a | n.a | n.a | n.a |
|  |  | **Eastern** | | | | | | | | | | | | | | | | | | | |
|  |  |  | Adigrat town | 2021/06/10 | 2021/06/17 | 101,242 | n.a | n.a | n.a | n.a | n.a | n.a | n.a | n.a | n.a | n.a | n.a | n.a | n.a | n.a | n.a |
|  |  | **Mekelle** | | | | | | | | | | | | | | | | | | | |
|  |  |  | Mekelle | 2021/06/10 | 2021/06/17 | 97,390 | n.a | n.a | n.a | n.a | n.a | n.a | n.a | n.a | n.a | n.a | n.a | n.a | n.a | n.a | n.a |
|  |  | **North Western** | | | | | | | | | | | | | | | | | | | |
|  |  |  | Asgede | 2021/06/10 | 2021/06/17 | 26,543 | n.a | n.a | n.a | n.a | n.a | n.a | n.a | n.a | n.a | n.a | n.a | n.a | n.a | n.a | n.a |
|  |  |  | Sheraro Town | 2021/06/10 | 2021/06/17 | 20,109 | n.a | n.a | n.a | n.a | n.a | n.a | n.a | n.a | n.a | n.a | n.a | n.a | n.a | n.a | n.a |
|  |  |  | Shire Town | 2021/06/10 | 2021/06/17 | 121,487 | n.a | n.a | n.a | n.a | n.a | n.a | n.a | n.a | n.a | n.a | n.a | n.a | n.a | n.a | n.a |
|  |  |  | Tahtay Koraro | 2021/06/10 | 2021/06/17 | 55,412 | n.a | n.a | n.a | n.a | n.a | n.a | n.a | n.a | n.a | n.a | n.a | n.a | n.a | n.a | n.a |
|  |  | **South Eastern** | | | | | | | | | | | | | | | | | | | |
|  |  |  | Degua Temben | 2021/06/10 | 2021/06/17 | 33,440 | n.a | n.a | n.a | n.a | n.a | n.a | n.a | n.a | n.a | n.a | n.a | n.a | n.a | n.a | n.a |
|  |  |  | Hintalo | 2021/06/10 | 2021/06/17 | 93,788 | n.a | n.a | n.a | n.a | n.a | n.a | n.a | n.a | n.a | n.a | n.a | n.a | n.a | n.a | n.a |
|  |  |  | Saharti | 2021/06/10 | 2021/06/17 | 85,878 | n.a | n.a | n.a | n.a | n.a | n.a | n.a | n.a | n.a | n.a | n.a | n.a | n.a | n.a | n.a |
|  |  | **Southern** | | | | | | | | | | | | | | | | | | | |
|  |  |  | Maichew Town | 2021/06/10 | 2021/06/17 | 34,842 | n.a | n.a | n.a | n.a | n.a | n.a | n.a | n.a | n.a | n.a | n.a | n.a | n.a | n.a | n.a |
| **Total-A4** | | | | **2021/06/10** | **2021/06/17** | **840,774** | **n.a** | **n.a** | **n.a** | **n.a** | **n.a** | **n.a** | **n.a** | **n.a** | **n.a** | **n.a** | **n.a** | **n.a** | **n.a** | **n.a** | **n.a** |
| **A5** | **Oromia** | | | | | | | | | | | | | | | | | | | | |
|  |  | **Bale** | | | | | | | | | | | | | | | | | | | |
|  |  |  | Berbere | 2021/12/23 | 2022/01/01 | 130,201 | 9,532 | 11,411 | 22,601 | 22,564 | 32,255 | 31,838 | 2022/03/25 | 2022/03/31 | 130,201 | 9,535 | 11,411 | 22,601 | 22,564 | 32,255 | 31,835 |
|  |  |  | D/menaa | 2021/12/23 | 2022/01/01 | 130,532 | 14,895 | 16,038 | 24,570 | 23,454 | 26,788 | 24,787 | 2022/03/25 | 2022/03/31 | 130,542 | 14,912 | 16,040 | 24,570 | 23,445 | 26,784 | 24,791 |
|  |  |  | H/Buluk | 2021/12/23 | 2022/01/01 | 117,349 | 11,642 | 11,964 | 23,185 | 22,377 | 23,961 | 24,220 | 2022/03/25 | 2022/03/31 | 117,345 | 11,642 | 11,964 | 23,185 | 22,377 | 23,961 | 24,216 |
|  |  |  | M/welabu | 2021/12/23 | 2022/01/01 | 138,752 | 12,890 | 13,575 | 22,796 | 24,869 | 33,205 | 31,417 | 2022/03/25 | 2022/03/31 | 138,746 | 12,886 | 13,575 | 22,796 | 24,869 | 33,205 | 31,415 |
|  | **Total** | | | **2021/12/23** | **2022/01/01** | **516,834** | **48,959** | **52,988** | **93,152** | **93,264** | **116,209** | **112,262** | **2022/03/25** | **2022/03/31** | **516,834** | **48,975** | **52,990** | **93,152** | **93,255** | **116,205** | **112,257** |
|  | **Somali** | | | | | | | | | | | | | | | | | | | | |
|  |  | **Liban** | | | | | | | | | | | | | | | | | | | |
|  |  |  | Bokolmayo | 2021/12/23 | 2022/01/01 | 51,093 | 3,769 | 3,395 | 8,582 | 16,991 | 2,047 | 16,309 | 2022/03/25 | 2022/03/31 | 51,093 | 3,773 | 3,395 | 8,582 | 16,991 | 2,047 | 16,305 |
|  |  |  | Deka Suftu | 2021/12/23 | 2022/01/01 | 58,989 | 8,346 | 6,597 | 12,035 | 10,737 | 13,216 | 8,058 | 2022/03/25 | 2022/03/31 | 58,987 | 8,346 | 6,597 | 12,035 | 10,737 | 13,216 | 8,056 |
|  |  |  | Dolo Odo | 2021/12/23 | 2022/01/01 | 142,068 | 13,848 | 9,239 | 26,022 | 32,327 | 29,852 | 30,780 | 2022/03/25 | 2022/03/31 | 142,064 | 13,848 | 9,239 | 26,022 | 32,327 | 29,852 | 30,776 |
|  |  |  | Filtu | 2021/12/23 | 2022/01/01 | 112,597 | 5,276 | 5,896 | 22,557 | 19,621 | 24,888 | 34,359 | 2022/03/25 | 2022/03/31 | 112,593 | 5,276 | 5,896 | 22,557 | 19,621 | 24,888 | 34,355 |
|  |  |  | Goro Baqaqsa | 2021/12/23 | 2022/01/01 | 67,149 | 8,113 | 8,711 | 13,449 | 14,023 | 11,423 | 11,430 | 2022/03/25 | 2022/03/31 | 67,148 | 8,113 | 8,711 | 13,449 | 14,023 | 11,425 | 11,427 |
|  |  |  | Gura Damole | 2021/12/23 | 2022/01/01 | 27,424 | 2,558 | 2,696 | 4,790 | 5,166 | 6,133 | 6,081 | 2022/03/25 | 2022/03/31 | 27,424 | 2,558 | 2,696 | 4,790 | 5,169 | 6,133 | 6,078 |
|  |  |  | Kersadula | 2021/12/23 | 2022/01/01 | 60,715 | 6,030 | 6,161 | 10,633 | 11,313 | 13,767 | 12,811 | 2022/03/25 | 2022/03/31 | 60,716 | 6,030 | 6,161 | 10,633 | 11,318 | 13,767 | 12,807 |
|  | **Total** | | | **2021/12/23** | **2022/01/01** | **520,035** | **47,940** | **42,695** | **98,068** | **110,178** | **101,326** | **119,828** | **2022/03/25** | **2022/03/31** | **520,025** | **47,944** | **42,695** | **98,068** | **110,186** | **101,328** | **119,804** |
| **Total-A5** | | | | **2021/12/23** | **2022/01/01** | **1,036,869** | **96,899** | **95,683** | **191,220** | **203,442** | **217,535** | **232,090** | **2022/03/25** | **2022/03/31** | **1,036,859** | **96,919** | **95,685** | **191,220** | **203,441** | **217,533** | **232,061** |
| **N/A^13^** | **Afar** | | | | | | | | | | | | | | | | | | | | |
|  |  | **Zone 1** | | | | | | | | | | | | | | | | | | | |
|  |  |  | Adaar | 2022/04/23 | 2022/04/30 | 12,886 | 1,399 | 1,149 | 2,113 | 1,561 | 3,834 | 2,830 | n.a | n.a | n.a | n.a | n.a | n.a | n.a | n.a | n.a |
|  |  |  | Chifra | 2022/04/23 | 2022/04/30 | 15,192 | 1,510 | 1,674 | 2,380 | 2,262 | 3,632 | 3,734 | n.a | n.a | n.a | n.a | n.a | n.a | n.a | n.a | n.a |
|  |  |  | Kori | 2022/04/23 | 2022/04/30 | 2,130 | 185 | 229 | 333 | 243 | 426 | 714 | n.a | n.a | n.a | n.a | n.a | n.a | n.a | n.a | n.a |
|  |  | **Zone 2** | | | | | | | | | | | | | | | | | | | |
|  |  |  | Abaala | 2022/04/23 | 2022/04/30 | 10,647 | 1,344 | 1,278 | 1,908 | 1,617 | 2,058 | 2,442 | n.a | n.a | n.a | n.a | n.a | n.a | n.a | n.a | n.a |
|  |  |  | Berahle | 2022/04/23 | 2022/04/30 | 6,120 | 527 | 493 | 1,111 | 1,121 | 1,133 | 1,735 | n.a | n.a | n.a | n.a | n.a | n.a | n.a | n.a | n.a |
|  |  |  | Erebti | 2022/04/23 | 2022/04/30 | 4,505 | 422 | 418 | 694 | 568 | 1,221 | 1,182 | n.a | n.a | n.a | n.a | n.a | n.a | n.a | n.a | n.a |
|  |  |  | Koneba | 2022/04/23 | 2022/04/30 | 7,244 | 1,118 | 1,072 | 1,232 | 1,370 | 456 | 1,996 | n.a | n.a | n.a | n.a | n.a | n.a | n.a | n.a | n.a |
|  |  |  | Megale | 2022/04/23 | 2022/04/30 | 9,813 | 701 | 623 | 2,201 | 2,323 | 1,953 | 2,012 | n.a | n.a | n.a | n.a | n.a | n.a | n.a | n.a | n.a |
|  |  | **Zone 3** | | | | | | | | | | | | | | | | | | | |
|  |  |  | Afdera | 2022/04/23 | 2022/04/30 | 2,738 | 339 | 241 | 419 | 305 | 880 | 554 | n.a | n.a | n.a | n.a | n.a | n.a | n.a | n.a | n.a |
|  |  | **Zone 4** | | | | | | | | | | | | | | | | | | | |
|  |  |  | Awra | 2022/04/23 | 2022/04/30 | 12,070 | 1,828 | 1,245 | 3,244 | 3,546 | 1,374 | 833 | n.a | n.a | n.a | n.a | n.a | n.a | n.a | n.a | n.a |
|  |  |  | Ewa | 2022/04/23 | 2022/04/30 | 12,012 | 900 | 894 | 1,863 | 1,905 | 3,242 | 3,208 | n.a | n.a | n.a | n.a | n.a | n.a | n.a | n.a | n.a |
|  |  |  | Golina | 2022/04/23 | 2022/04/30 | 12,535 | 1,654 | 1,224 | 2,240 | 1,822 | 3,038 | 2,557 | n.a | n.a | n.a | n.a | n.a | n.a | n.a | n.a | n.a |
|  |  |  | Teru | 2022/04/23 | 2022/04/30 | 13,529 | 280 | 241 | 661 | 601 | 6,256 | 5,490 | n.a | n.a | n.a | n.a | n.a | n.a | n.a | n.a | n.a |
|  |  |  | Yalo | 2022/04/23 | 2022/04/30 | 5,966 | 456 | 382 | 1,043 | 890 | 1,591 | 1,604 | n.a | n.a | n.a | n.a | n.a | n.a | n.a | n.a | n.a |
|  |  | **Zone 5** | | | | | | | | | | | | | | | | | | | |
|  |  |  | Dewe | 2022/04/23 | 2022/04/30 | 8,083 | 872 | 910 | 1,768 | 1,537 | 1,498 | 1,498 | n.a | n.a | n.a | n.a | n.a | n.a | n.a | n.a | n.a |
|  |  |  | Hadalela | 2022/04/23 | 2022/04/30 | 9,288 | 424 | 362 | 1,541 | 1,410 | 2,866 | 2,685 | n.a | n.a | n.a | n.a | n.a | n.a | n.a | n.a | n.a |
|  |  |  | Samurobi | 2022/04/23 | 2022/04/30 | 7,664 | 731 | 742 | 1,407 | 1,441 | 1,786 | 1,557 | n.a | n.a | n.a | n.a | n.a | n.a | n.a | n.a | n.a |
|  |  |  | Telalak | 2022/04/23 | 2022/04/30 | 7,918 | 862 | 849 | 1,304 | 1,382 | 1,644 | 1,877 | n.a | n.a | n.a | n.a | n.a | n.a | n.a | n.a | n.a |
|  | **Total** | | | **2022/04/23** | **2022/04/30** | **160,340** | **15,552** | **14,026** | **27,462** | **25,904** | **38,888** | **38,508** | **n.a** | **n.a** | **n.a** | **n.a** | **n.a** | **n.a** | **n.a** | **n.a** | **n.a** |
|  | **Amhara** | | | | | | | | | | | | | | | | | | | | |
|  |  | **Waghimra** | | | | | | | | | | | | | | | | | | | |
|  |  |  | Dehana | 2022/05/23 | 2022/05/31 | 64,343 | 6,635 | 7,251 | 10,924 | 12,682 | 11,464 | 15,387 | n.a | n.a | n.a | n.a | n.a | n.a | n.a | n.a | n.a |
|  |  |  | Gazgibla | 2022/05/23 | 2022/05/31 | 20,653 | 2,466 | 2,506 | 3,670 | 3,742 | 3,820 | 4,449 | n.a | n.a | n.a | n.a | n.a | n.a | n.a | n.a | n.a |
|  |  |  | Sahala | 2022/05/23 | 2022/05/31 | 17,128 | 1,728 | 1,724 | 2,796 | 3,158 | 3,213 | 4,509 | n.a | n.a | n.a | n.a | n.a | n.a | n.a | n.a | n.a |
|  |  |  | Sekota Town | 2022/05/23 | 2022/05/31 | 45,322 | 2,761 | 2,788 | 5,301 | 7,085 | 12,795 | 14,592 | n.a | n.a | n.a | n.a | n.a | n.a | n.a | n.a | n.a |
|  |  |  | Sekota Town (IDP^14^) | 2022/05/23 | 2022/05/31 | 13,723 | 1,319 | 1,821 | 1,226 | 1,515 | 3,543 | 4,299 | n.a | n.a | n.a | n.a | n.a | n.a | n.a | n.a | n.a |
|  |  |  | Sekota Zuria | 2022/05/23 | 2022/05/31 | 68,221 | 4,929 | 5,243 | 11,836 | 12,238 | 16,139 | 17,836 | n.a | n.a | n.a | n.a | n.a | n.a | n.a | n.a | n.a |
|  |  |  | Sekota Zuria (IDP) | 2022/05/23 | 2022/05/31 | 2,961 | 351 | 265 | 457 | 370 | 644 | 874 | n.a | n.a | n.a | n.a | n.a | n.a | n.a | n.a | n.a |
|  |  |  | Ziqwala | 2022/05/23 | 2022/05/31 | 32,837 | 2,719 | 3,016 | 4,342 | 5,149 | 7,695 | 9,916 | n.a | n.a | n.a | n.a | n.a | n.a | n.a | n.a | n.a |
|  | **Total** | | | **2022/05/23** | **2022/05/31** | **265,188** | **22,908** | **24,614** | **40,552** | **45,939** | **59,313** | **71,862** | **n.a** | **n.a** | **n.a** | **n.a** | **n.a** | **n.a** | **n.a** | **n.a** | **n.a** |
| **Total- vaccination using retrieved vaccines** | | | | **n.a** | **n.a** | **425,528** | **38,460** | **38,640** | **68,014** | **71,843** | **98,201** | **110,370** | **n.a** | **n.a** | **n.a** | **n.a** | **n.a** | **n.a** | **n.a** | **n.a** | **n.a** |
| **A6** | **Oromia** | | | | | | | | | | | | | | | | | | | | |
|  |  | **Bale** | | | | | | | | | | | | | | | | | | | |
|  |  |  | Berbere^15^ | 2023/01/13 | 2023/01/20 | 21,351 | 1,758 | 1,710 | 3,794 | 3,902 | 4,774 | 5,413 | n.a | n.a | n.a | n.a | n.a | n.a | n.a | n.a | n.a |
|  |  |  | Goro | 2023/01/13 | 2023/01/20 | 54,875 | 5,586 | 6,099 | 9,648 | 10,624 | 11,282 | 11,636 | n.a | n.a | n.a | n.a | n.a | n.a | n.a | n.a | n.a |
|  | **Total** | | | **2023/01/13** | **2023/01/20** | **76,226** | **7,344** | **7,809** | **13,442** | **14,526** | **16,056** | **17,049** | **n.a** | **n.a** | **n.a** | **n.a** | **n.a** | **n.a** | **n.a** | **n.a** | **n.a** |
|  | **Somali** | | | | | | | | | | | | | | | | | | | | |
|  |  | **Liban** | | | | | | | | | | | | | | | | | | | |
|  |  |  | Bokolmayo (IDP) | 2023/01/13 | 2023/01/20 | 8,196 | 906 | 921 | 1,762 | 1,854 | 1,244 | 1,509 | n.a | n.a | n.a | n.a | n.a | n.a | n.a | n.a | n.a |
|  |  |  | Dolo Ado | 2023/01/13 | 2023/01/20 | 16,291 | 2,050 | 1,989 | 2,674 | 2,847 | 3,420 | 3,311 | n.a | n.a | n.a | n.a | n.a | n.a | n.a | n.a | n.a |
|  | **Total** | | | **2023/01/13** | **2023/01/20** | **24,487** | **2,956** | **2,910** | **4,436** | **4,701** | **4,664** | **4,820** | **n.a** | **n.a** | **n.a** | **n.a** | **n.a** | **n.a** | **n.a** | **n.a** | **n.a** |
| **Total-A6** | | | | **2023/01/13** | **2023/01/20** | **100,713** | **10,300** | **10,719** | **17,878** | **19,227** | **20,720** | **21,869** | **n.a** | **n.a** | **n.a** | **n.a** | **n.a** | **n.a** | **n.a** | **n.a** | **n.a** |
| **A7** | **Oromia** | | | | | | | | | | | | | | | | | | | | |
|  |  | **Bale** | | | | | | | | | | | | | | | | | | | |
|  |  |  | Berbere | 2023/05/15 | 2023/05/24 | 126,331 | 8,271 | 9,390 | 20,605 | 21,912 | 33,121 | 33,032 | n.a | n.a | n.a | n.a | n.a | n.a | n.a | n.a | n.a |
|  |  |  | Goro | 2023/05/15 | 2023/05/24 | 113,589 | 8,500 | 9,528 | 18,543 | 18,535 | 29,651 | 28,832 | n.a | n.a | n.a | n.a | n.a | n.a | n.a | n.a | n.a |
|  |  |  | Gura Damole | 2023/05/15 | 2023/05/24 | 40,225 | 3,651 | 3,020 | 4,519 | 6,613 | 11,672 | 10,750 | n.a | n.a | n.a | n.a | n.a | n.a | n.a | n.a | n.a |
|  |  | **Borana** | | | | | | | | | | | | | | | | | | | |
|  |  |  | Moyale | 2023/05/15 | 2023/05/24 | 305,157 | 21,410 | 22,893 | 49,754 | 49,178 | 80,161 | 81,761 | n.a | n.a | n.a | n.a | n.a | n.a | n.a | n.a | n.a |
|  |  | **East Bale** | | | | | | | | | | | | | | | | | | | |
|  |  |  | Dawe Kachen | 2023/05/15 | 2023/05/24 | 42,677 | 3,267 | 4,170 | 5,589 | 7,801 | 11,289 | 10,561 | n.a | n.a | n.a | n.a | n.a | n.a | n.a | n.a | n.a |
|  |  |  | Ginir | 2023/05/15 | 2023/05/24 | 169,467 | 11,521 | 12,712 | 28,194 | 27,628 | 44,310 | 45,102 | n.a | n.a | n.a | n.a | n.a | n.a | n.a | n.a | n.a |
|  |  |  | Ginir Town | 2023/05/15 | 2023/05/24 | 54,195 | 5,017 | 4,070 | 7,318 | 8,846 | 14,219 | 14,725 | n.a | n.a | n.a | n.a | n.a | n.a | n.a | n.a | n.a |
|  |  |  | Raitu | 2023/05/15 | 2023/05/24 | 48,892 | 3,812 | 3,917 | 8,319 | 8,354 | 10,271 | 14,219 | n.a | n.a | n.a | n.a | n.a | n.a | n.a | n.a | n.a |
|  |  | **Guji** | | | | | | | | | | | | | | | | | | | |
|  |  |  | Girja | 2023/05/15 | 2023/05/24 | 69,394 | 4,198 | 5,205 | 10,972 | 11,313 | 19,346 | 18,360 | n.a | n.a | n.a | n.a | n.a | n.a | n.a | n.a | n.a |
|  |  |  | Goro Dola | 2023/05/15 | 2023/05/24 | 78,065 | 6,091 | 5,859 | 11,721 | 12,734 | 20,569 | 21,091 | n.a | n.a | n.a | n.a | n.a | n.a | n.a | n.a | n.a |
|  |  |  | Liben | 2023/05/15 | 2023/05/24 | 98,266 | 7,129 | 7,373 | 16,023 | 15,876 | 26,891 | 24,974 | n.a | n.a | n.a | n.a | n.a | n.a | n.a | n.a | n.a |
|  |  |  | Negele Town | 2023/05/15 | 2023/05/24 | 59,552 | 5,541 | 4,470 | 9,714 | 9,011 | 15,615 | 15,201 | n.a | n.a | n.a | n.a | n.a | n.a | n.a | n.a | n.a |
|  |  |  | Wadera | 2023/05/15 | 2023/05/24 | 67,163 | 5,120 | 5,042 | 10,957 | 9,500 | 17,823 | 18,721 | n.a | n.a | n.a | n.a | n.a | n.a | n.a | n.a | n.a |
|  |  | **West Arsi** | | | | | | | | | | | | | | | | | | | |
|  |  |  | Nensebo | 2023/05/15 | 2023/05/24 | 193,725 | 15,091 | 14,535 | 31,589 | 30,719 | 50,700 | 51,091 | n.a | n.a | n.a | n.a | n.a | n.a | n.a | n.a | n.a |
|  | **Total** | | | **2023/05/15** | **2023/05/24** | **1,466,698** | **108,619** | **112,184** | **233,817** | **238,020** | **385,638** | **388,420** | **n.a** | **n.a** | **n.a** | **n.a** | **n.a** | **n.a** | **n.a** | **n.a** | **n.a** |
|  | **Somali** | | | | | | | | | | | | | | | | | | | | |
|  |  | **Dawa** | | | | | | | | | | | | | | | | | | | |
|  |  |  | Moyale | 2023/05/15 | 2023/05/24 | 266,229 | 19,523 | 19,971 | 43,916 | 44,738 | 68,267 | 69,814 | n.a | n.a | n.a | n.a | n.a | n.a | n.a | n.a | n.a |
|  |  | **Liban** | | | | | | | | | | | | | | | | | | | |
|  |  |  | Dollo Ado | 2023/05/15 | 2023/05/24 | 120,804 | 9,812 | 9,064 | 19,699 | 18,250 | 32,316 | 31,663 | n.a | n.a | n.a | n.a | n.a | n.a | n.a | n.a | n.a |
|  |  |  | Guradamole | 2023/05/15 | 2023/05/24 | 55,674 | 4,017 | 4,176 | 7,911 | 9,170 | 15,812 | 14,588 | n.a | n.a | n.a | n.a | n.a | n.a | n.a | n.a | n.a |
|  | **Total** | | | **2023/05/15** | **2023/05/24** | **442,707** | **33,352** | **33,211** | **71,526** | **72,158** | **116,395** | **116,065** | **n.a** | **n.a** | **n.a** | **n.a** | **n.a** | **n.a** | **n.a** | **n.a** | **n.a** |
| **Total-A7** | | | | **2023/05/15** | **2023/05/24** | **1,909,405** | **141,971** | **145,395** | **305,343** | **310,178** | **502,033** | **504,485** | **n.a** | **n.a** | **n.a** | **n.a** | **n.a** | **n.a** | **n.a** | **n.a** | **n.a** |
| **A8** | **Oromia** | | | | | | | | | | | | | | | | | | | | |
|  |  | **West Guji** | | | | | | | | | | | | | | | | | | | |
|  |  |  | Bule Hora | 2023/08/10 | 2023/08/20 | 286,802 | 23,938 | 27,886 | 53,912 | 48,412 | 64,949 | 67,705 | n.a | n.a | n.a | n.a | n.a | n.a | n.a | n.a | n.a |
|  |  |  | Dugda Dawa | 2023/08/10 | 2023/08/20 | 99,320 | 13,998 | 14,322 | 15,880 | 16,317 | 18,279 | 20,524 | n.a | n.a | n.a | n.a | n.a | n.a | n.a | n.a | n.a |
|  |  |  | Melka Soda | 2023/08/10 | 2023/08/20 | 84,456 | 9,331 | 9,451 | 11,605 | 13,397 | 19,702 | 20,970 | n.a | n.a | n.a | n.a | n.a | n.a | n.a | n.a | n.a |
|  |  |  | Liben Chukala | 2023/08/10 | 2023/08/20 | 100,524 | 10,277 | 10,699 | 16,315 | 17,132 | 23,822 | 22,279 | n.a | n.a | n.a | n.a | n.a | n.a | n.a | n.a | n.a |
|  | **Total** | | | **2023/08/10** | **2023/08/20** | **571,102** | **57,544** | **62,358** | **97,712** | **95,258** | **126,752** | **131,478** | **n.a** | **n.a** | **n.a** | **n.a** | **n.a** | **n.a** | **n.a** | **n.a** | **n.a** |
|  | **Sidama** | | | | | | | | | | | | | | | | | | | | |
|  |  | **Hawassa** | | | | | | | | | | | | | | | | | | | |
|  |  |  | Hawassa Zuria | 2023/08/10 | 2023/08/20 | 70,277 | 6,080 | 6,593 | 13,871 | 15,214 | 13,481 | 15,038 | n.a | n.a | n.a | n.a | n.a | n.a | n.a | n.a | n.a |
|  |  |  | Hawela | 2023/08/10 | 2023/08/20 | 148,234 | 19,803 | 20,744 | 23,778 | 25,071 | 27,845 | 30,993 | n.a | n.a | n.a | n.a | n.a | n.a | n.a | n.a | n.a |
|  |  | **Sidama** | | | | | | | | | | | | | | | | | | | |
|  |  |  | Dara | 2023/08/10 | 2023/08/20 | 124,645 | 17,372 | 17,647 | 19,952 | 20,035 | 23,542 | 26,097 | n.a | n.a | n.a | n.a | n.a | n.a | n.a | n.a | n.a |
|  | **Total** | | | **2023/08/10** | **2023/08/20** | **343,156** | **43,255** | **44,984** | **57,601** | **60,320** | **64,868** | **72,128** | **n.a** | **n.a** | **n.a** | **n.a** | **n.a** | **n.a** | **n.a** | **n.a** | **n.a** |
|  | **SNNPR** | | | | | | | | | | | | | | | | | | | | |
|  |  | **Alle Special** | | | | | | | | | | | | | | | | | | | |
|  |  |  | Alle Special | 2023/08/10 | 2023/08/20 | 88,527 | 9,621 | 9,972 | 14,376 | 15,738 | 18,647 | 20,173 | n.a | n.a | n.a | n.a | n.a | n.a | n.a | n.a | n.a |
|  |  | **Amaro Special** | | | | | | | | | | | | | | | | | | | |
|  |  |  | Amaro | 2023/08/10 | 2023/08/20 | 161,961 | 12,166 | 11,466 | 24,142 | 26,441 | 44,094 | 43,652 | n.a | n.a | n.a | n.a | n.a | n.a | n.a | n.a | n.a |
|  |  | **Burji Special** | | | | | | | | | | | | | | | | | | | |
|  |  |  | Burji Special | 2023/08/10 | 2023/08/20 | 80,235 | 8,702 | 9,228 | 12,698 | 13,647 | 18,208 | 17,752 | n.a | n.a | n.a | n.a | n.a | n.a | n.a | n.a | n.a |
|  |  | **Gamo** | | | | | | | | | | | | | | | | | | | |
|  |  |  | Arba Minch Zuria | 2023/08/10 | 2023/08/20 | 251,749 | 31,773 | 32,233 | 40,291 | 41,421 | 51,490 | 54,541 | n.a | n.a | n.a | n.a | n.a | n.a | n.a | n.a | n.a |
|  |  | **Gedeo** | | | | | | | | | | | | | | | | | | | |
|  |  |  | Sawla Town | 2023/08/10 | 2023/08/20 | 48,289 | 2,183 | 3,455 | 7,705 | 9,703 | 11,523 | 13,720 | n.a | n.a | n.a | n.a | n.a | n.a | n.a | n.a | n.a |
|  |  | **Gofa** | | | | | | | | | | | | | | | | | | | |
|  |  |  | Denba Gofa | 2023/08/10 | 2023/08/20 | 102,842 | 8,159 | 8,723 | 17,581 | 17,630 | 25,037 | 25,712 | n.a | n.a | n.a | n.a | n.a | n.a | n.a | n.a | n.a |
|  |  | **Hadiya** | | | | | | | | | | | | | | | | | | | |
|  |  |  | Gerese | 2023/08/10 | 2023/08/20 | 128,111 | 6,662 | 8,721 | 20,804 | 22,853 | 32,524 | 36,547 | n.a | n.a | n.a | n.a | n.a | n.a | n.a | n.a | n.a |
|  |  | **Konso** | | | | | | | | | | | | | | | | | | | |
|  |  |  | Karat Zuria | 2023/08/10 | 2023/08/20 | 131,938 | 12,027 | 12,663 | 22,475 | 23,506 | 26,928 | 34,339 | n.a | n.a | n.a | n.a | n.a | n.a | n.a | n.a | n.a |
|  |  |  | Kena | 2023/08/10 | 2023/08/20 | 79,070 | 6,292 | 6,583 | 13,176 | 13,734 | 16,243 | 23,042 | n.a | n.a | n.a | n.a | n.a | n.a | n.a | n.a | n.a |
|  |  |  | Segen Zuria | 2023/08/10 | 2023/08/20 | 48,338 | 4,963 | 4,923 | 10,278 | 10,242 | 8,594 | 9,338 | n.a | n.a | n.a | n.a | n.a | n.a | n.a | n.a | n.a |
|  |  | **South Omo** | | | | | | | | | | | | | | | | | | | |
|  |  |  | Bena Tsemay | 2023/08/10 | 2023/08/20 | 75,113 | 7,664 | 7,629 | 13,463 | 13,180 | 14,876 | 18,301 | n.a | n.a | n.a | n.a | n.a | n.a | n.a | n.a | n.a |
|  |  |  | Malie | 2023/08/10 | 2023/08/20 | 119,510 | 11,857 | 12,192 | 20,293 | 21,419 | 25,918 | 27,831 | n.a | n.a | n.a | n.a | n.a | n.a | n.a | n.a | n.a |
|  | **Total** | | | **2023/08/10** | **2023/08/20** | **1,315,683** | **122,069** | **127,788** | **217,282** | **229,514** | **294,082** | **324,948** | **n.a** | **n.a** | **n.a** | **n.a** | **n.a** | **n.a** | **n.a** | **n.a** | **n.a** |
| **Total-A8** | | | | **2023/08/10** | **2023/08/20** | **2,229,941** | **222,868** | **235,130** | **372,595** | **385,092** | **485,702** | **528,554** | **n.a** | **n.a** | **n.a** | **n.a** | **n.a** | **n.a** | **n.a** | **n.a** | **n.a** |
| **A9** | **Amhara** | | | | | | | | | | | | | | | | | | | | |
|  |  | **Bahir Dar City** | | | | | | | | | | | | | | | | | | | |
|  |  |  | Bahir Dar Town | 2023/09/16 | 2023/09/23 | 368,649 | 37,872 | 37,872 | 64,983 | 67,370 | 85,807 | 74,745 | n.a | n.a | n.a | n.a | n.a | n.a | n.a | n.a | n.a |
|  |  | **Central Gonder** | | | | | | | | | | | | | | | | | | | |
|  |  |  | East Dembia | 2023/09/16 | 2023/09/23 | 172,533 | 17,299 | 17,299 | 29,409 | 32,139 | 40,788 | 35,599 | n.a | n.a | n.a | n.a | n.a | n.a | n.a | n.a | n.a |
|  |  | **Debre Birhan Town** | | | | | | | | | | | | | | | | | | | |
|  |  |  | Debre Berhan Town | 2023/09/16 | 2023/09/23 | 151,406 | 15,143 | 15,143 | 26,744 | 27,259 | 35,830 | 31,287 | n.a | n.a | n.a | n.a | n.a | n.a | n.a | n.a | n.a |
|  |  | **Gondar City** | | | | | | | | | | | | | | | | | | | |
|  |  |  | Gondar Town | 2023/09/16 | 2023/09/23 | 368,270 | 37,615 | 37,602 | 63,902 | 67,707 | 86,414 | 75,030 | n.a | n.a | n.a | n.a | n.a | n.a | n.a | n.a | n.a |
|  |  | **North Gojam** | | | | | | | | | | | | | | | | | | | |
|  |  |  | Bahirdar Zuria | 2023/09/16 | 2023/09/23 | 193,564 | 19,394 | 19,394 | 32,970 | 36,410 | 45,607 | 39,789 | n.a | n.a | n.a | n.a | n.a | n.a | n.a | n.a | n.a |
|  |  | **North Shewa** | | | | | | | | | | | | | | | | | | | |
|  |  |  | Mojan Wedera | 2023/09/16 | 2023/09/23 | 74,100 | 7,418 | 7,418 | 12,611 | 13,353 | 17,663 | 15,637 | n.a | n.a | n.a | n.a | n.a | n.a | n.a | n.a | n.a |
|  |  | **South Gonder** | | | | | | | | | | | | | | | | | | | |
|  |  |  | Dera (AM) | 2023/09/16 | 2023/09/23 | 261,789 | 26,203 | 26,203 | 45,545 | 47,165 | 62,267 | 54,406 | n.a | n.a | n.a | n.a | n.a | n.a | n.a | n.a | n.a |
|  |  | **West Gondar** | | | | | | | | | | | | | | | | | | | |
|  |  |  | Metema | 2023/09/16 | 2023/09/23 | 139,028 | 13,911 | 13,911 | 23,649 | 25,740 | 32,995 | 28,822 | n.a | n.a | n.a | n.a | n.a | n.a | n.a | n.a | n.a |
|  |  |  | Quara | 2023/09/16 | 2023/09/23 | 129,133 | 12,932 | 12,932 | 22,384 | 24,277 | 29,744 | 26,864 | n.a | n.a | n.a | n.a | n.a | n.a | n.a | n.a | n.a |
| **Total-A9** | | | | **2023/09/16** | **2023/09/23** | **1,858,472** | **187,787** | **187,774** | **322,197** | **341,420** | **437,115** | **382,179** | **n.a** | **n.a** | **n.a** | **n.a** | **n.a** | **n.a** | **n.a** | **n.a** | **n.a** |
| **A10** | **Afar** | | | | | | | | | | | | | | | | | | | | |
|  |  | **Zone 3** | | | | | | | | | | | | | | | | | | | |
|  |  |  | Amibara | 2023/11/11 | 2023/11/21 | 93,418 | 9,350 | 9,350 | 15,895 | 17,617 | 21,506 | 19,700 | n.a | n.a | n.a | n.a | n.a | n.a | n.a | n.a | n.a |
|  |  |  | Awash | 2023/11/11 | 2023/11/21 | 146,328 | 14,635 | 14,935 | 24,881 | 26,944 | 33,662 | 31,271 | n.a | n.a | n.a | n.a | n.a | n.a | n.a | n.a | n.a |
|  |  |  | Awash Town | 2023/11/11 | 2023/11/21 | 69,774 | 6,987 | 6,987 | 12,878 | 12,877 | 16,071 | 13,974 | n.a | n.a | n.a | n.a | n.a | n.a | n.a | n.a | n.a |
|  |  |  | Dulecha | 2023/11/11 | 2023/11/21 | 93,026 | 9,319 | 9,319 | 16,842 | 16,774 | 21,834 | 18,938 | n.a | n.a | n.a | n.a | n.a | n.a | n.a | n.a | n.a |
|  |  |  | Hanruka | 2023/11/11 | 2023/11/21 | 40,697 | 4,093 | 4,093 | 6,941 | 7,968 | 9,415 | 8,187 | n.a | n.a | n.a | n.a | n.a | n.a | n.a | n.a | n.a |
|  |  | **Zone 4** | | | | | | | | | | | | | | | | | | | |
|  |  |  | Teru | 2023/11/11 | 2023/11/21 | 80,930 | 8,105 | 8,605 | 13,779 | 14,589 | 18,642 | 17,210 | n.a | n.a | n.a | n.a | n.a | n.a | n.a | n.a | n.a |
|  | **Total** | | | **2023/11/11** | **2023/11/21** | **524,173** | **52,489** | **53,289** | **91,216** | **96,769** | **121,130** | **109,280** | **n.a** | **n.a** | **n.a** | **n.a** | **n.a** | **n.a** | **n.a** | **n.a** | **n.a** |
|  | **Sidama** | | | | | | | | | | | | | | | | | | | | |
|  |  | **Sidama** | | | | | | | | | | | | | | | | | | | |
|  |  |  | Dara Otilicho | 2023/11/11 | 2023/11/21 | 110,705 | 11,082 | 11,782 | 18,840 | 19,948 | 25,889 | 23,164 | n.a | n.a | n.a | n.a | n.a | n.a | n.a | n.a | n.a |
|  |  |  | Wondo-Genet | 2023/11/11 | 2023/11/21 | 197,709 | 19,776 | 19,976 | 33,620 | 36,998 | 45,786 | 41,553 | n.a | n.a | n.a | n.a | n.a | n.a | n.a | n.a | n.a |
|  |  |  | Wondo-Genet Town | 2023/11/11 | 2023/11/21 | 56,856 | 5,689 | 5,689 | 9,472 | 10,841 | 13,986 | 11,179 | n.a | n.a | n.a | n.a | n.a | n.a | n.a | n.a | n.a |
|  | **Total** | | | **2023/11/11** | **2023/11/21** | **365,270** | **36,547** | **37,447** | **61,932** | **67,787** | **85,661** | **75,896** | **n.a** | **n.a** | **n.a** | **n.a** | **n.a** | **n.a** | **n.a** | **n.a** | **n.a** |
|  | **SNNPR** | | | | | | | | | | | | | | | | | | | | |
|  |  | **Gamo** | | | | | | | | | | | | | | | | | | | |
|  |  |  | Arba Minch Zuria | 2023/11/11 | 2023/11/21 | 127,899 | 12,806 | 12,806 | 21,770 | 23,951 | 29,654 | 26,912 | n.a | n.a | n.a | n.a | n.a | n.a | n.a | n.a | n.a |
|  |  | **Gedeo** | | | | | | | | | | | | | | | | | | | |
|  |  |  | Chelelektu Town | 2023/11/11 | 2023/11/21 | 57,609 | 5,776 | 5,776 | 9,820 | 11,398 | 13,286 | 11,553 | n.a | n.a | n.a | n.a | n.a | n.a | n.a | n.a | n.a |
|  |  |  | Dila Town | 2023/11/11 | 2023/11/21 | 96,183 | 9,631 | 9,631 | 16,772 | 17,336 | 22,551 | 20,262 | n.a | n.a | n.a | n.a | n.a | n.a | n.a | n.a | n.a |
|  |  | **Gofa** | | | | | | | | | | | | | | | | | | | |
|  |  |  | Zala | 2023/11/11 | 2023/11/21 | 104,199 | 10,928 | 10,728 | 17,929 | 18,771 | 23,986 | 21,857 | n.a | n.a | n.a | n.a | n.a | n.a | n.a | n.a | n.a |
|  |  | **Gurage** | | | | | | | | | | | | | | | | | | | |
|  |  |  | Sodo | 2023/11/11 | 2023/11/21 | 247,074 | 24,722 | 24,722 | 43,827 | 44,999 | 57,860 | 50,944 | n.a | n.a | n.a | n.a | n.a | n.a | n.a | n.a | n.a |
|  | **Total** | | | **2023/11/11** | **2023/11/21** | **632,964** | **63,863** | **63,663** | **110,118** | **116,455** | **147,337** | **131,528** | **n.a** | **n.a** | **n.a** | **n.a** | **n.a** | **n.a** | **n.a** | **n.a** | **n.a** |
| **Total-A10** | | | | **2023/11/11** | **2023/11/21** | **1,522,407** | **152,899** | **154,399** | **263,266** | **281,011** | **354,128** | **316,704** | **n.a** | **n.a** | **n.a** | **n.a** | **n.a** | **n.a** | **n.a** | **n.a** | **n.a** |
| **A11** | **Amhara** | | | | | | | | | | | | | | | | | | | | |
|  |  | **North Wollo** | | | | | | | | | | | | | | | | | | | |
|  |  |  | Gazgibla | 2023/11/29 | 2023/12/05 | 40,386 | 2,450 | 2,349 | 5,118 | 5,791 | 11,868 | 12,810 | n.a | n.a | n.a | n.a | n.a | n.a | n.a | n.a | n.a |
|  |  | **Waghimra** | | | | | | | | | | | | | | | | | | | |
|  |  |  | Kobo Town | 2023/11/29 | 2023/12/05 | 90,417 | 2,706 | 3,214 | 14,919 | 15,012 | 27,567 | 26,999 | n.a | n.a | n.a | n.a | n.a | n.a | n.a | n.a | n.a |
|  | **Total** | | | **2023/11/29** | **2023/12/05** | **130,803** | **5,156** | **5,563** | **20,037** | **20,803** | **39,435** | **39,809** | **n.a** | **n.a** | **n.a** | **n.a** | **n.a** | **n.a** | **n.a** | **n.a** | **n.a** |
|  | **Oromia** | | | | | | | | | | | | | | | | | | | | |
|  |  | **East Hararge** | | | | | | | | | | | | | | | | | | | |
|  |  |  | Bedeno | 2023/11/29 | 2023/12/05 | 56,172 | 4,815 | 5,085 | 10,691 | 11,847 | 12,098 | 11,636 | n.a | n.a | n.a | n.a | n.a | n.a | n.a | n.a | n.a |
|  |  |  | Girawa | 2023/11/29 | 2023/12/05 | 60,504 | 4,924 | 5,313 | 10,089 | 10,246 | 14,734 | 15,198 | n.a | n.a | n.a | n.a | n.a | n.a | n.a | n.a | n.a |
|  |  |  | Gole Oda | 2023/11/29 | 2023/12/05 | 52,043 | 4,379 | 5,032 | 10,233 | 10,534 | 10,669 | 11,196 | n.a | n.a | n.a | n.a | n.a | n.a | n.a | n.a | n.a |
|  |  |  | Haromaya | 2023/11/29 | 2023/12/05 | 81,463 | 8,561 | 8,575 | 16,006 | 15,112 | 15,694 | 17,515 | n.a | n.a | n.a | n.a | n.a | n.a | n.a | n.a | n.a |
|  |  |  | Kumbi | 2023/11/29 | 2023/12/05 | 51,552 | 4,574 | 4,971 | 9,295 | 9,620 | 9,933 | 13,159 | n.a | n.a | n.a | n.a | n.a | n.a | n.a | n.a | n.a |
|  |  |  | Kurfa Chele | 2023/11/29 | 2023/12/05 | 84,860 | 6,144 | 6,416 | 15,229 | 14,564 | 20,525 | 21,982 | n.a | n.a | n.a | n.a | n.a | n.a | n.a | n.a | n.a |
|  |  |  | Kersa | 2023/11/29 | 2023/12/05 | 87,948 | 6,100 | 6,620 | 17,719 | 15,991 | 21,595 | 19,923 | n.a | n.a | n.a | n.a | n.a | n.a | n.a | n.a | n.a |
|  |  |  | Mayu Muluke | 2023/11/29 | 2023/12/05 | 66,806 | 7,674 | 8,857 | 10,056 | 11,497 | 12,406 | 16,316 | n.a | n.a | n.a | n.a | n.a | n.a | n.a | n.a | n.a |
|  |  | **West Guji** | | | | | | | | | | | | | | | | | | | |
|  |  |  | Abaya | 2023/11/29 | 2023/12/05 | 99,785 | 10,401 | 10,531 | 17,087 | 17,292 | 21,410 | 23,064 | n.a | n.a | n.a | n.a | n.a | n.a | n.a | n.a | n.a |
|  |  |  | Gelana | 2023/11/29 | 2023/12/05 | 90,390 | 7,207 | 8,205 | 15,101 | 15,354 | 21,571 | 22,952 | n.a | n.a | n.a | n.a | n.a | n.a | n.a | n.a | n.a |
|  | **Total** | | | **2023/11/29** | **2023/12/05** | **731,523** | **64,779** | **69,605** | **131,506** | **132,057** | **160,635** | **172,941** | **n.a** | **n.a** | **n.a** | **n.a** | **n.a** | **n.a** | **n.a** | **n.a** | **n.a** |
| **Total-A11** | | | | **2023/11/29** | **2023/12/05** | **862,326** | **69,935** | **75,168** | **151,543** | **152,860** | **200,070** | **212,750** | **n.a** | **n.a** | **n.a** | **n.a** | **n.a** | **n.a** | **n.a** | **n.a** | **n.a** |
| **Total-A1 to A11 (including vaccination using retrieved vaccines)** | | | | **n.a** | **n.a** | **13,911,605** | **1,139,236** | **1,164,940** | **2,217,962** | **2,306,471** | **3,120,028** | **3,122,194** | **n.a** | **n.a** | **4,002,915** | **304,379** | **305,114** | **685,885** | **712,173** | **979,517** | **1,015,847** |
| **Grand Total** | | | | **n.a** | **n.a** | **14,215,949** | **1,150,706** | **1,176,867** | **2,236,509** | **2,325,331** | **3,137,986** | **3,144,990** | **n.a** | **n.a** | **4,103,848** | **316,033** | **317,931** | **704,111** | **730,962** | **997,606** | **1,037,205** |

**Footnote:**

^1^ Request number refers to each OCV request made by the Ethiopian government.

^2^ Zone is the second administrative division in Ethiopia, subdivided from Region.

^3^ Woreda is the third administrative division in Ethiopia, subdivided from Zone.

^4^ In first round.

^5^ 12-59 months.

^6^ 60-179 months.

^7^ Bilateral: Ethiopian government’s bilateral request to the government of the Republic of Korea for OCV doses for cholera outbreak control in 2019.

^8^ SNNPR: Southern Nations, Nationalities, and Peoples’ Region

^9^ n.a: not applicable.

^10^ ECCP: ‘Ethiopia Cholera Control and Prevention’ Project

^11^ One of the homeless shelters in Addis Ababa.

^12^ IPDC: Industrial Park Development Corporation.

^13^ It used remaining OCV doses from previous OCV vaccination campaigns.^14^ IDP: Internally Displaced People

^15^ Target population included community and IDP.
